# Thermodynamics and Kinetics of the Deintercalation of a Novel Anthracycline from Double-Stranded Oligonucleotide DNA

**DOI:** 10.1021/acs.jpcb.5c03021

**Published:** 2025-08-12

**Authors:** Georgios Mikaelian, Haralambos Sarimveis, Doros N. Theodorou, Grigorios Megariotis

**Affiliations:** † School of Chemical Engineering, 68994 National Technical University of Athens (NTUA), 9 Heroon Polytechniou Street, Zografou Campus, Athens, GR 15780, Greece; ‡ Academy of Athens, 28 Panepistimiou Street, Athens, GR 10679, Greece; § School of Engineering, Department of Mineral Resources Engineering, University of Western Macedonia, Kozani 50100, Greece

## Abstract

We present an *in silico* study of the deintercalation of berubicin from two double-stranded oligonucleotide DNA sequences using well-tempered metadynamics (WT-MetaD), an enhanced sampling method widely employed for biomolecular systems. In our recent study ( MikaelianG.,  J. Phys. Chem. B 2024, 128­(26), 6291–6307 38899795 10.1021/acs.jpcb.4c02213PMC11228990), the thermodynamics of DNA–berubicin complexes in the intercalated state was examined in detail. Continuing this study here, we focus on the thermodynamics and kinetics of deintercalation, undertaking a rigorous analysis of individual stages in its mechanism. The Gibbs energy surface is first calculated as a function of two collective variables (used in prior studies of anthracyclines), followed by an estimation of deintercalation times. These deintercalation times are comparable to—or even exceed—those of clinically established anthracyclines in widespread therapeutic use. Our simulations reveal that the deintercalation mechanism involves two stable states—the intercalated state and the minor groove-bound state—as well as a reshuffling state. This three-step mechanism that emerges is supported by other published studies concerning DNA–anthracycline complexes. The rate-limiting step of the deintercalation mechanism is the transition from the intercalated to the reshuffling state, and the applied method allows a detailed description of it. Each state is described in terms of various properties, whose values are consistent with published computational and experimental studies on the interactions of other anthracyclines with DNA.

## Graphical abstract

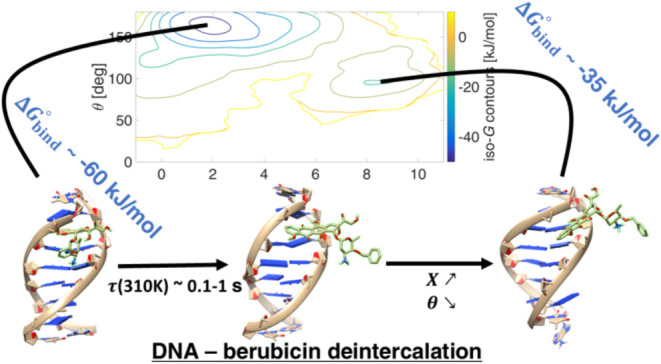

## 1 Introduction

Anthracyclines are a well-known class of anticancer drugs employed for the treatment of various types of cancer.^1^ In general, anthracyclines are not able to cross the blood brain barrier^2,3^ and thus are not administered for malignant tumors in the central nervous system.^4^ Berubicin, the drug under consideration in this study, is an anthracycline, yet has the ability to cross the blood-brain barrier.^4–9^ It is currently undergoing clinical trials (phase II)^10^ and is very promising for the challenging treatment of glioblastoma multiforme, one of the most aggressive brain tumors.

In a recent article of ours,^11^ noncovalent DNA–berubicin intercalation complexes were studied computationally in water, with particular attention to the rigorous thermodynamic description of the binding process, as well as to structure and geometry in the bound state. The thermodynamic analysis was mainly based on the double decoupling method^12–15^ (DDM) and the solvent balance method^16^ (SBM), and in that context, the entropic and enthalpic contributions to the standard binding Gibbs energy were estimated. Herein, our focus is mainly on the deintercalation process of berubicin from oligonucleotide DNA sequences, with the analysis addressing both thermodynamics and kinetics; it is pointed out that a mere thermodynamic study of the binding and unbinding process is not sufficient. In the pharmacology of receptor–ligand complexes, the measurement of equilibrium properties (such as the binding free energy, the half maximal inhibitory concentration (IC_50_), etc.) without consideration of kinetic properties may lead to an inaccurate assessment of the *in vivo* effectiveness.^17–21^ Deintercalation is considered a process of tremendous significance and its kinetic analysis is directly related to the pharmacological efficacy of drugs.^22–27^ Moreover, a systematic investigation of the deintercalation kinetics may reveal important details of the underlying mechanism and its individual stages. In view of this, and taking into account that a drug exerts its pharmacological action only when bound to its receptor,^18,28^ ref 11 and the present article complement one another. Also, some stages of the deintercalation mechanism may exhibit pharmacological action, and their stabilization potentially enhances the therapeutic effect.^29,30^ Wherever feasible, we critically discuss our kinetic and thermodynamic findings in view of related published results concerning doxorubicin and daunorubicin, which are prototype drugs of the anthracycline class. To the best of our knowledge, there are no systematic studies on berubicin, and, for this reason, a comprehensive comparison with other anthracyclines in clinical use is made in the present article.

Molecular dynamics is a powerful classical simulation method broadly employed for the investigation of chemical and biomolecular systems, such as lipid membranes, DNA, RNA, etc.^31–33^ However, many phenomena, especially those of particular biological interest, take place at time scales longer than those that can be addressed by conventional full-atom or united-atom molecular dynamics simulations. To meet this challenge while retaining atomistic resolution, enhanced sampling methods may be applied that properly sample specific regions of the phase space. Such a method is umbrella sampling,^34–36^ which has been used widely for the estimation of thermodynamic properties through calculation of the potential of mean force (PMF) along a selected reaction coordinate which is usually characteristic of the geometry of the system. The method in question has been extensively applied for small organic molecules in biomolecular systems, including drugs acting on the central nervous system and neurotransmitters (e.g., dopamine).^37–43^ Another popular enhanced sampling method is metadynamics, which has been widely employed for biomolecular systems and especially those consisting of proteins and/or DNA.^29,44–63^ In the framework of metadynamics, a history-dependent potential, which biases the time evolution of the system, is constructed as a sum of Gaussian functions placed along the trajectory in a space defined by a few collective variables (CVs) that are utilized for the description of the slow modes of the system.^64^ In cases where the definition of CVs is straightforward, the computation of the related free energy surface (either Gibbs energy or Helmholtz energy) is, in principle, feasible, with its basins playing a crucial role in the thermodynamic analysis. In this article, we make use of well-tempered metadynamics^65,66^ (WT-MetaD), in the context of which the standard metadynamics serves as a limiting case. WT-MetaD is first applied for the thermodynamic description of the considered systems through construction of the Gibbs energy surface. Next, a detailed kinetic analysis is carried out in terms of rate constants, investigation of the individual stages of the deintercalation mechanism and the deintercalation time. The Gaussian functions used in WT-MetaD serve to extract the system from deep free energy wells in which it is mostly trapped when DNA and berubicin are found in the bound state. By applying this method, the transition from the bound to the unbound state takes place on a time scale of nanoseconds. Subsequently, making use of transition state theory in the framework of WT-MetaD, we extract the actual dynamics of the deintercalation process. The aforementioned combined computational scheme is applied as detailed in refs 67,68 . Our simulations identify three deintercalation states: two stable states (intercalated and minor groove-bound) and one reshuffling state. We also observe longer deintercalation times than those of other anthracyclines.

The article is organized as follows. In Section 2, a detailed description of all the employed methods and models is provided. Section 3 presents the simulation results along with a full discussion of them in terms of selected thermodynamic and kinetic properties. The last section summarizes the most important conclusions deduced from the research undertaken.

## 2 Models and Methods

The deintercalation of berubicin from DNA in a water environment is investigated *in silico* with the drug molecule being considered only in the protonated state, as shown in Figure 1; at a pH of 7.2, most of berubicin is found in that state.^11^ The two DNA structures investigated herein are those reported in ref 11, i.e.,: 5′-d­(AC|GTAC|GT)-3′ and 5′-d­(T|GT|ACA)-3′, both obtained from the Protein Data Bank under the code numbers 1AL9 ^69^ and 1AMD,^69^ respectively. In these sequences, the symbol | shows where the intercalation sites are located. The molecular docking simulations of ref 11 revealed that the most stable intercalation sites are 5′-d­(ACGTAC|GT)-3′ and 5′-d­(TGT|ACA)-3′ for 5′-d­(AC|GTAC|GT)-3′ and 5′-d­(T|GT|ACA)-3′, respectively. For this reason, only the latter set of intercalation sites is taken into account in this article. The simulation details (force fields, equilibration protocol and the parameters used for the molecular dynamics simulations) are exactly the same as those reported in ref 11 (Section 2 therein). Each molecular dynamics simulation conducted in the context of this article has a duration of approximately 100–150 ns. The initial configurations for these simulations are representative configurations of DNA–berubicin complexes in the bound state, as obtained from the simulations of ref 11. PLUMED (PLUgin for MolEcular Dynamics) is an open-source code^70–72^ for the implementation of metadynamics, including the WT-MetaD method, which is employed in this article. In addition, PLUMED is interfaced with GROMACS,^73^ which is employed for the molecular dynamics simulations of all the systems under consideration herein. More specifically, our results are generated using a combination of GROMACS 2021.4 and PLUMED 2.7.2.

**Figure 1:**
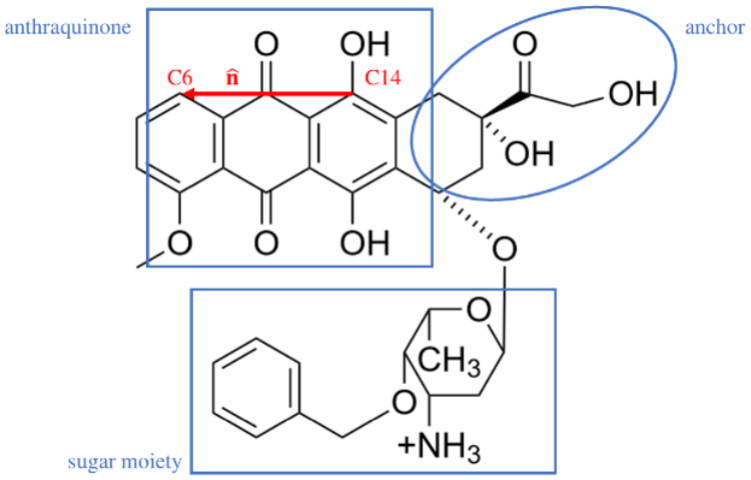
Molecular structure of the protonated berubicin.

The CVs must be selected such that they change significantly during deintercalation. In this article, two CVs are used: a distance metric and an angular parameter, both adapted from earlier studies of DNA–ligand intercalation complexes.^29,44–46,54,55,63,74^ It is noteworthy that in several of these references the ligand is an anthracycline.^29,74^ For a better understanding of the definition of CVs, the reader is referred to Figure 2, which provides a schematic representation of the 5′-d­(ACGTAC|GT)-3′–berubicin complex. An analogous representation for 5′-d­(TGT|ACA)-3′–berubicin is given in Figure S1 of the Supporting Information. Prior to the definition of the CVs, we define some geometric elements. **IS** stands for the position vector of the center of mass (COM) of the two consecutive DNA base pairs that delineate the intercalation site. More precisely, this COM for 5′-d­(ACGTAC|GT)-3′ is defined by the following nucleotides: C10, G11, C6 and G7, which are also labeled in Figure 2. Concerning the 5′-d­(TGT|ACA)-3′–berubicin complex, the COM is calculated by considering the T9, A10, T3 and A4 nucleotides, which are highlighted in Figure S1. **MG** represents the position vector of the COM of the deoxyriboses belonging to two of the four complementary nucleotides of the intercalation site (their atoms are highlighted as spheres in Figures 2 and S1) that lie toward the minor groove, and **L** represents the position vector of the COM of berubicin. Next, a body-fixed unit vector in the DNA molecule, **m̂**, is defined by the vector connecting **IS** and **MG**, with **MG** representing the end point of the vector. Another vector needed is **d = L – IS**. As for berubicin, a unit vector **n̂** is defined along the direction connecting atoms C14 and C6 (marked in Figure 1), with the latter being the end point of **n̂**. This vector is representative of the orientation of the anthraquinone group of berubicin. It is remarked that anthraquinone is buried in the intercalation site.^11^ After all the above definitions, the first CV is the projection of **d** onto **m̂**, namely *X* **= m̂•d**. The second CV is defined as the angle formed between **m̂** and **n̂**, namely *θ* = cos^–1^ (**m̂•n̂)**.

**Figure 2:**
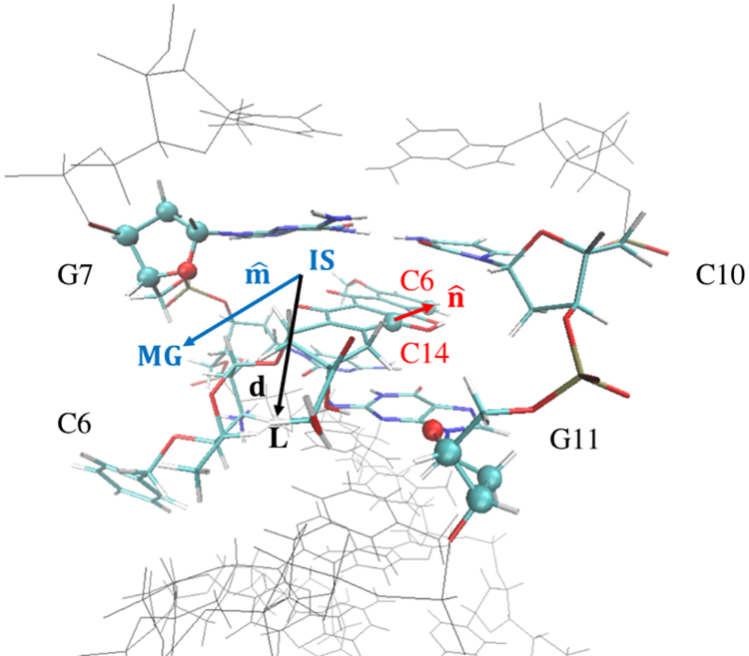
Schematic representation of the points and vectors employed for the definition of the two CVs for the 5′-d­(ACGTAC|GT)-3′–berubicin complex.

A short description of WT-MetaD is provided in Section S3 of the Supporting Information, while all the necessary parameters used in PLUMED are listed in Table 1. A brief discussion of the choice of these parameters is given in the Supporting Information (Section S7). For each of the two systems studied in this article, multiple independent molecular dynamics simulations are performed to ensure the proper application of WT-MetaD. The results presented in the next section represent averages over *N*_run_ simulations unless specified otherwise. From a thermodynamic perspective, the characteristic function of the systems being examined is the Gibbs energy, since all the reported molecular dynamics simulations are carried out in the isothermal–isobaric statistical ensemble. In the general two-dimensional case, the following equation is employed for the standard binding Gibbs energy:

1 $$\Delta G_{\mathrm{bind}}^{{}^{\circ}}=-\mathrm{RT}\cdot \ln\left\{\frac{\iint_{\left(b\right)}\exp\left[-\frac{G\left(X,\theta \right)}{\mathrm{RT}}\right]dXd\theta }{\iint_{\left(\mathrm{ub}\right)}\exp\left[-\frac{G\left(X,\theta \right)}{\mathrm{RT}}\right]dXd\theta }\right\}-\mathrm{RT}\cdot \ln\left(\frac{C^{◦}}{C_{\mathrm{site}}}\right)$$

*C*° stands for the standard concentration (1 M), and *C*_site_ is the concentration of the binding sites in the system. The integrals in eq 1 are computed over the region of the CV space that corresponds to the bound state (b) and the unbound state (ub). The derivation of eq 1 is presented in Section S4, and its implementation is carried out using an in-house computational code, where all necessary numerical integrations are performed using the trapezoidal rule. The convergence tests, as well as an error analysis, are given in Section S5.

**Table 1:** List of the WT-MetaD Parameters Used in PLUMED^a^

*h*_0_	0.25 kJ/mol
Δ*t*	2 ps
Δ*T*	5115 K
σ_X_	0.25 Å
σ_θ_	2.06°
*N*_run_	6

a The parameters in the first column are described in Section S7 of the Supporting Information.

## 3 Results and Discussion

Figure 3 depicts the two Gibbs energy surfaces; the corresponding Gibbs energy contour plots over the CV space are displayed in Figure 4, in which the value of the free energy for each iso-*G* is given. The latter plots provide more distinct information about the minima. It is worth mentioning that the Gibbs energy surfaces are shifted by an energy constant so as to be close to zero in the unbound region. In all cases, two deep basins are observed, separated by a wide plateau on the Gibbs energy surface. In Figure 4b there is a third Gibbs energy basin at low *X* and θ values, but this corresponds to a nonstandard interaction between DNA and berubicin and is therefore not considered in our analysis. To aid in understanding the plots in Figure 4, black circles are used to mark the relevant Gibbs energy minima whose coordinates are listed in Table 2. An important conclusion drawn from the aforementioned table is that the minima of each complex are in close proximity, corresponding to the same bound states, and they are both close to the ones in ref 29. In all simulations, the complexes spend some time in the intercalated state and, after passing through a reshuffling state, are found in the minor groove-bound state. For more details about the reshuffling state, the reader is referred to Section S3 of the Supporting Information. The transition point appears as an abrupt change in the plots of the acceleration factor, α­(*t*), versus simulation time, as clearly seen in Figures 5 and S2, which pertain to indicative simulation runs of the 5′-d­(ACGTAC|GT)-3′–berubicin and 5′-d­(TGT|ACA)-3′–berubicin complexes. According to Tiwary et al.,^67^ a kinetic transition must correspond to such an abrupt kink in α­(*t*), as mentioned in Section S3. The transition points on the CV space are reported in Table 2 and marked with red crosses in Figure 4, indicating transitions from the intercalated state to the reshuffling state. Their standard errors are shown along the ***X*** and ***θ*** axes. After a cluster analysis, representative configurations of the 5′-d­(ACGTAC|GT)-3′–berubicin and 5′-d­(TGT|ACA)-3′–berubicin complexes in the intercalated, reshuffling and minor groove-bound state are visualized in Figures 6 and S3, respectively. DNA and berubicin pass through these states sequentially, and they finally end up in the unbound state. This indicates a three-step deintercalation mechanism for both the DNA–berubicin complexes, which is also observed for a DNA–daunorubicin complex.^27,29^

**Figure 3:**
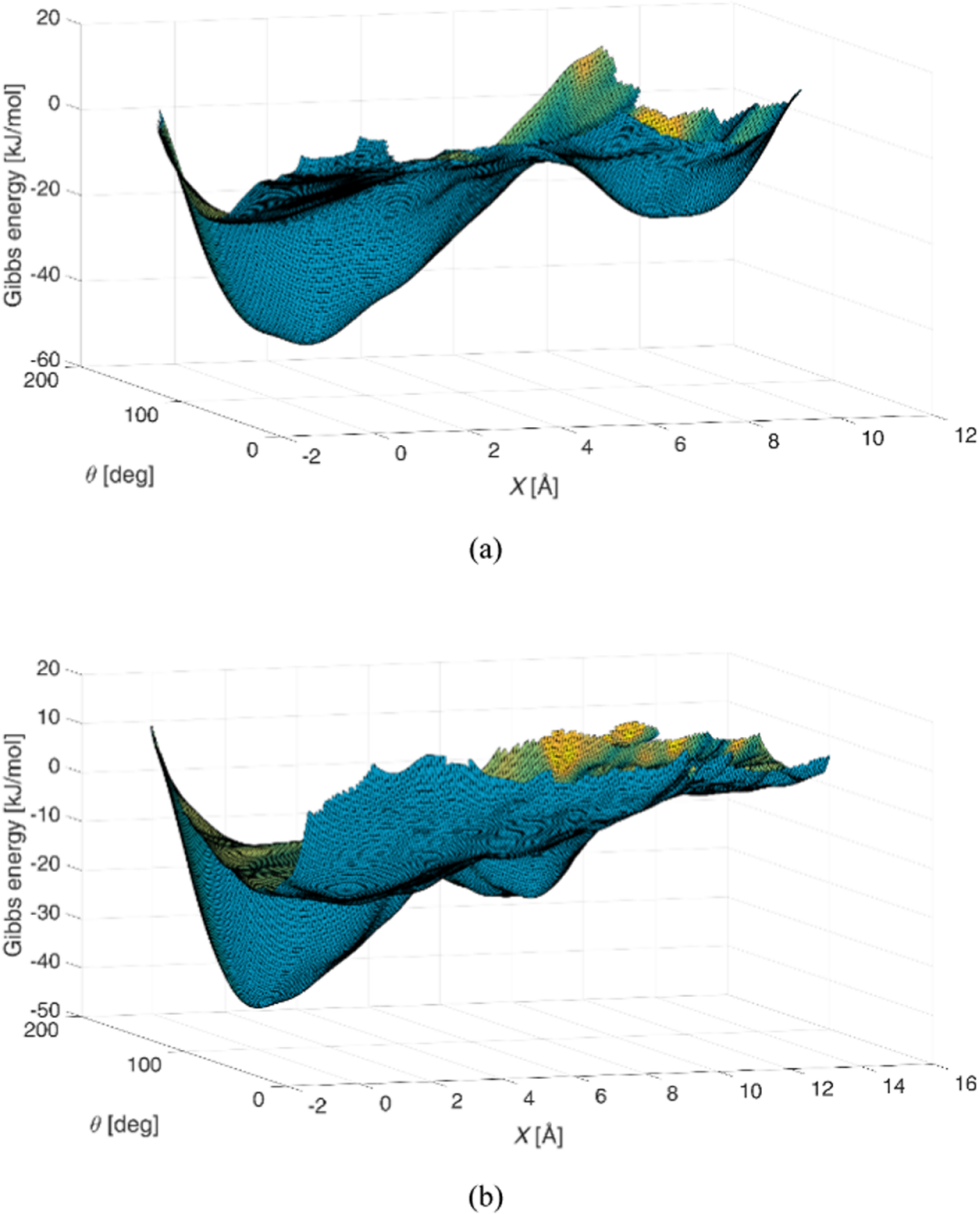
Gibbs energy surfaces as functions of the two CVs for (a) 5′-d­(ACGTAC|GT)-3′–berubicin and (b) 5′-d­(TGT|ACA)-3′–berubicin.

**Figure 4:**
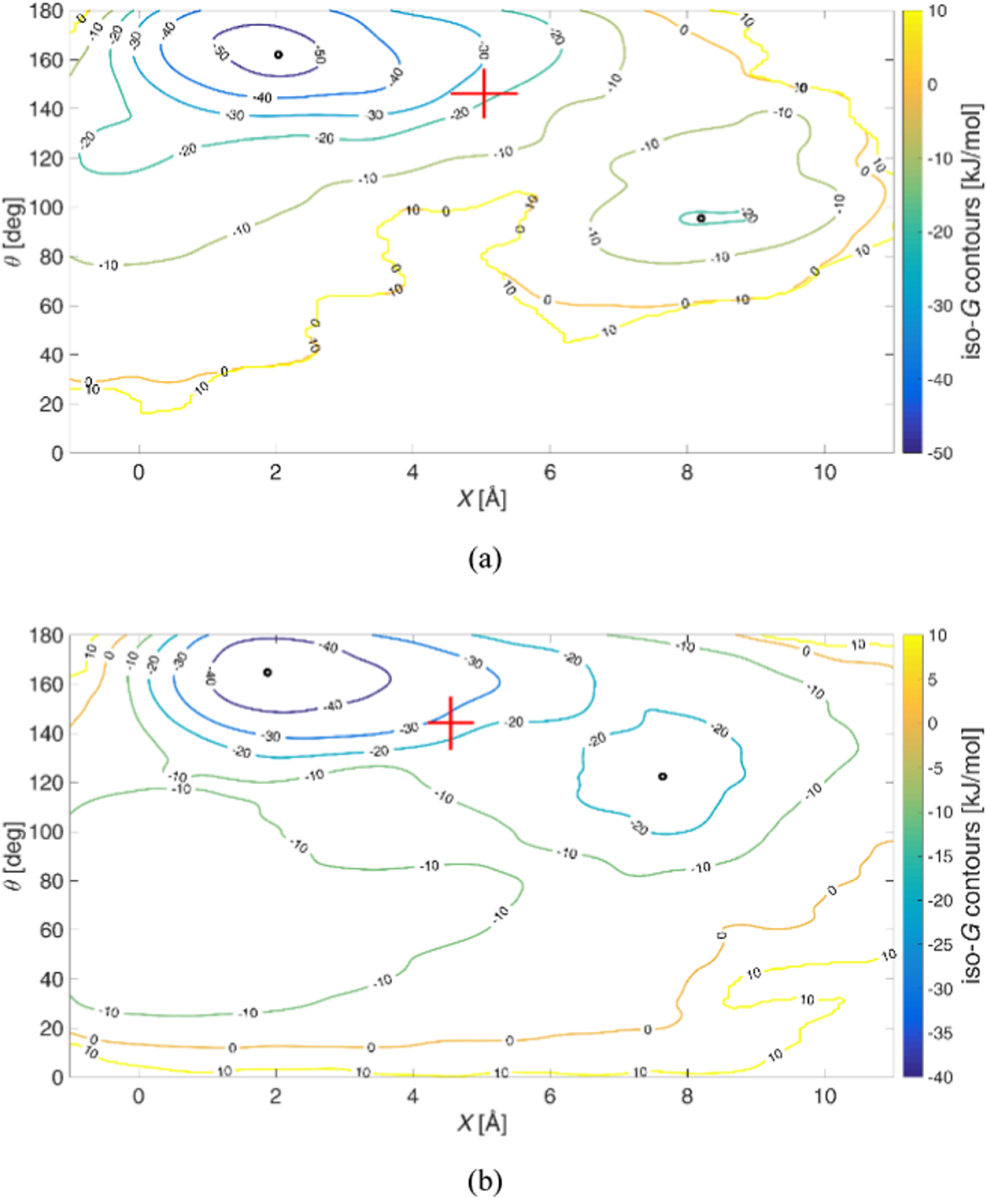
Gibbs energy contours over the CV space for (a) 5′-d­(ACGTAC|GT)-3′–berubicin and (b) 5′-d­(TGT|ACA)-3′–berubicin. The Gibbs energy basins and transition states are depicted with black circles and red crosses, respectively.

**Figure 5:**
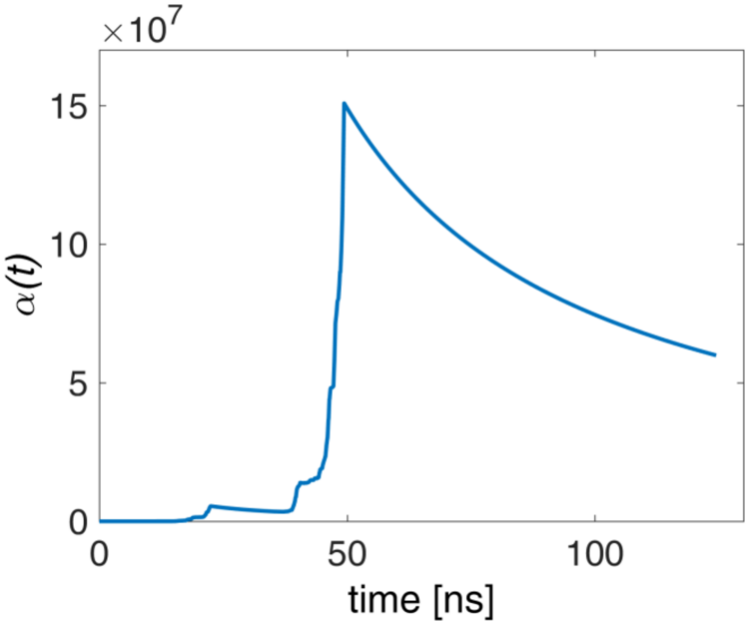
Acceleration factor as a function of simulation time for the 5′-d­(ACGTAC|GT)-3′–berubicin complex.

**Figure 6:**
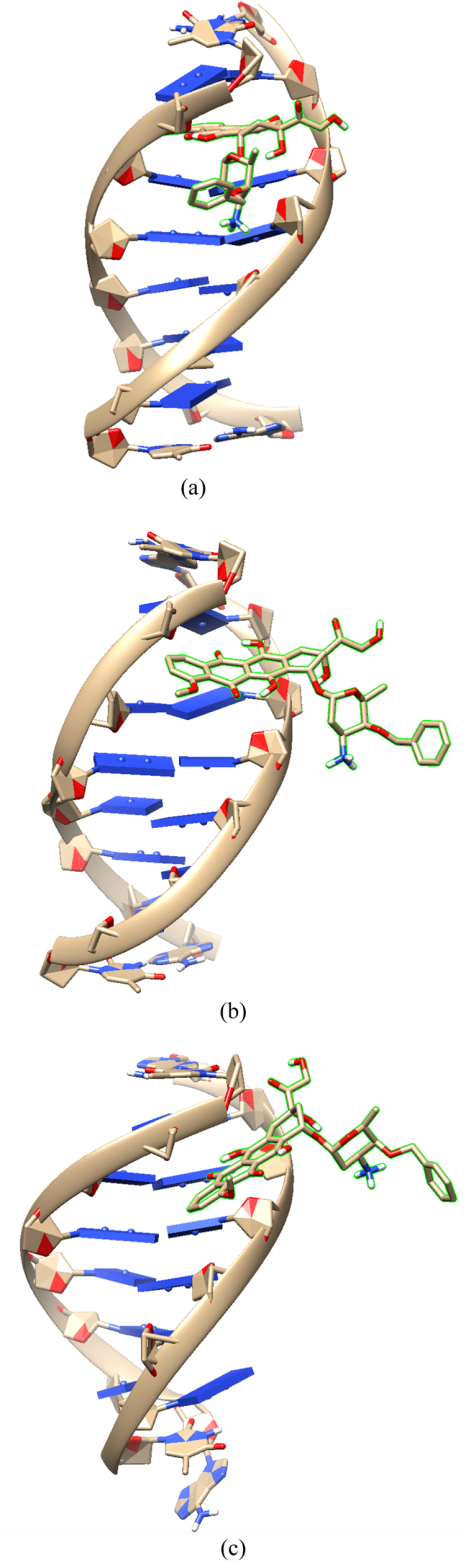
Representative configurations of the 5′-d­(ACGTAC|GT)-3′–berubicin complexes during the transition from the intercalated state to the minor groove-bound state. (a) intercalated state, (b) reshuffling state and (c) minor groove-bound state.

**Table 2:** Coordinates of the Minima and Transition Points of the Gibbs Energy Surfaces^a^,^b^

system	CV_min1_ [Å, deg]	CV_min2_ [Å, deg]	CV_trans_ [Å,deg]
5′-d(ACGTAC|GT)-3′–berubicin	(2.04, 162.0)	(8.20, 95.4)	(5.04, 146.2)
5′-d(TGT|ACA)-3′–berubicin	(1.88, 164.7)	(7.64, 122.4)	(4.55 144.1)

a min and trans correspond to the minima and transition points, respectively.

b 1 and 2 indicate the first and second minimum, corresponding to the intercalated and the minor groove-bound states, respectively.

Moreover, the time evolution of the two CVs is provided in Figure 7, which refers to a single representative run of the 5′-d­(ACGTAC|GT)-3′–berubicin complex. The vertical black lines in Figure 7 delineate the transitions under consideration: (1) from the intercalated to the reshuffling state, (2) from the reshuffling to the minor groove-bound state and (3) from the minor groove-bound to the unbound state. It is noteworthy that a number of related articles have also identified these bound states for DNA–anthracycline complexes.^27,29,75^ In the first region of Figure 7, the small and positive values of ***X*** are interpreted by a dual mode of interaction between DNA and berubicin: the anthraquinone planar structure of berubicin is intercalated into the DNA sequence, and simultaneously, the sugar moiety of berubicin is located in the minor groove of the DNA sequence. This finding is in full agreement with published articles concerning DNA and anthracyclines,^29,74,76–90^ including ref 11 which focuses on berubicin–DNA complexes. In the intercalated state, *X* takes values up to 4–5 Å, whereas θ is close to 180° because the anthraquinone moiety is oriented parallel to the DNA bases, and the methoxy group is positioned toward the major groove. This has also been observed in the literature on the DNA–anthracycline complexes.^11,81,91^ During reshuffling, the gradual detachment of the drug from the intercalated state is accompanied by a breakdown of the intercalation site. The existence of this state throughout deintercalation suggests that the formation of the intercalation site in the first place is a drug-induced process, namely the intercalation site is formed once the drug is externally bound with DNA. This is not consistent with the natural fluctuation hypothesis for the intercalation site formation,^55,92^ which states that the intercalation site is spontaneously formed in the unbound state and the drug diffuses into it. This finding is in agreement with published articles on DNA–drug intercalations, including DNA–anthracycline ones.^27,55,76^ Generalizing this trend, such a slow drug-induced reshuffling state is present in most binding mechanisms of high-affinity target–ligand complexes.^17,18,28^ Regarding the minor groove-bound state, both DNA sequences adopt conformations very similar to those of B-DNA in water, as stated in the related literature on DNA–ligand minor groove-bound complexes, including DNA–anthracycline ones.^29,51,93–96^ DNA oligomers adopt the B-DNA form when they are free in water.^97^ In the minor groove-bound state, the reported values for *X* range from 7 to 9 Å, whereas the θ values are significantly lower than 180°. To sum up, these geometric considerations provide insight into DNA–berubicin intercalation, the inverse process of deintercalation. More specifically, the formation of the intercalated state is described by the drug-induced cavity formation model, in which the intercalation site is formed once berubicin is bound to DNA. In contrast, the minor groove-bound state generally follows the lock-and-key model: berubicin binds to DNA maintained in its natural B-DNA conformation in aqueous solution. It is worth examining the one-dimensional PMF as a function of *X* by integrating out θ. Figure 8 depicts the PMFs for the two DNA–berubicin complexes. At small values of *X*, a deep basin is observed in both curves, while at larger values of *X*, a shallow basin emerges at higher PMF values. These correspond to the intercalated and minor groove-bound states, respectively.

**Figure 7:**
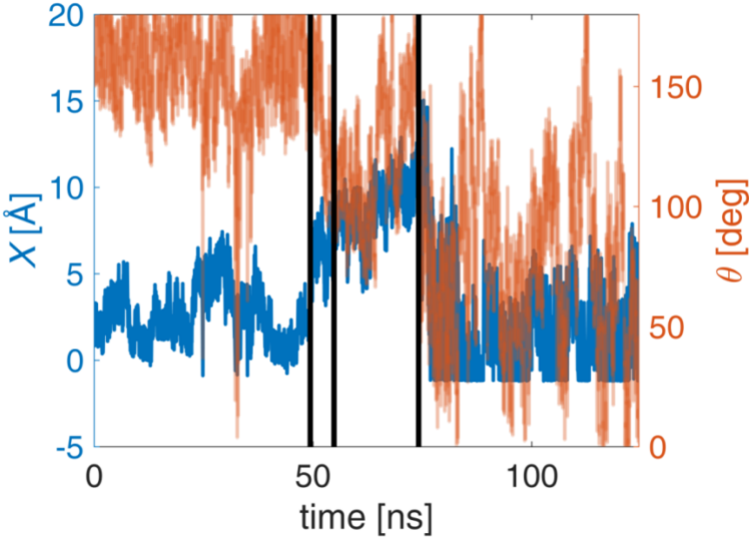
Time evolution of the two CVs in the case of 5′-d­(ACGTAC|GT)-3′–berubicin. The vertical black lines mark the transitions during deintercalation, as detailed in the text.

**Figure 8:**
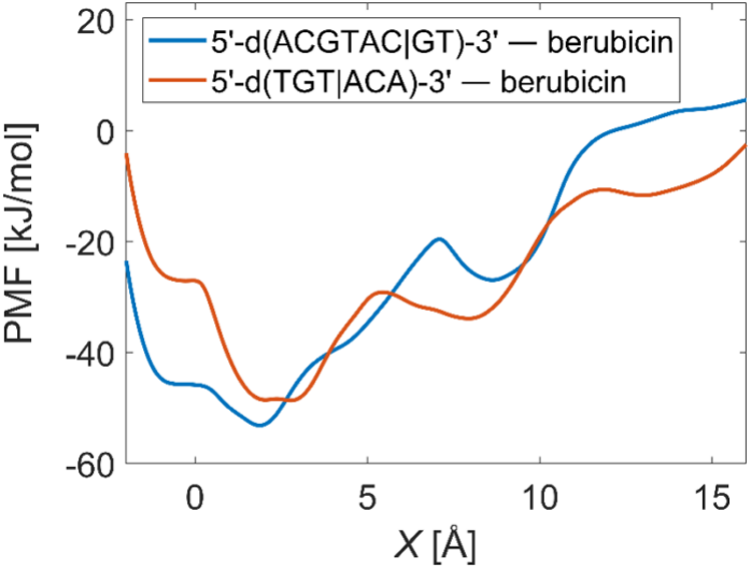
One-dimensional PMF for the deintercalation of each of the two DNA–berubicin complexes under investigation.

The first thermodynamic property calculated in the framework of our analysis is the standard Gibbs energy change for the transfer of berubicin from the unbound state to the two stable bound states detailed in the previous paragraph. The thermodynamic calculations are performed by applying eq 1 and the results are summarized in Table 3. First, it is worth noting the strong agreement between the binding Gibbs energies in the intercalated state for the two DNA sequences and those reported in ref 11, where the standard binding Gibbs energies of the intercalated states were calculated using the DDM method. The observed agreement between the two methods for estimating binding Gibbs energies supports the proper implementation of WT-MetaD in the complex systems studied herein. The binding Gibbs energies for the two DNA–berubicin complexes are quite similar and are higher than those of other anthracyclines complexed with DNA, such as doxorubicin and daunorubicin. The reader is referred to ref 11 and some references therein for direct comparison purposes. Another important conclusion drawn from Table 3 is that the minor groove-bound state is less favorable, from a thermodynamic point of view, compared to the intercalated state; this finding is also reported in published *in silico* and experimental studies involving other anthracyclines interacting with DNA.^27,29,74–77,98^ Quantitatively, the binding Gibbs energies for the minor groove-bound state fall in the wide range of −24 to −42 kJ/mol.^27,29,74,75,77,98^

**Table 3:** Standard Binding Gibbs Energies for the Two Stable Bound States in Both DNA–Berubicin Complexes^a^,^b^

system	Δ*G*_int_ [kJ/mol]	Δ*G*_mg_ [kJ/mol]
5′-d(ACGTAC|GT)-3′–berubicin	–63.2 ± 1.7 (−63.2)	–31.5 ± 1.7
5′-d(TGT|ACA)-3′–berubicin	–60.4 ± 2.8 (−65.7)	–38.5 ± 2.8

a The indices *int* and *mg* stand for the intercalated and minor groove-bound state, respectively.

b The values in parentheses are taken from ref 11.

The analysis proceeds with the calculation of mean (natural) residence times, τ, whose reciprocals correspond to the associated kinetic constants. We focus only on the first stage of the deintercalation process, i.e., the change from the intercalated state to the reshuffling state. The reason is that the aforementioned change is fully described by the two selected CVs that satisfy the conditions set by Tiwary and Parrinello,^67^ leading to a well-behaved Poisson distribution of residence times. From a qualitative point of view, the other stages are brief compared to the first stage, which is the rate-limiting step of the deintercalation mechanism, as also supported by other DNA–anthracyclines deintercalations.^27,75,77,98,99^ For this reason, the mean residence time in the intercalated state effectively corresponds to the deintercalation time.^27^ Next, cumulative distribution functions of the residence time are constructed from the multiple runs conducted for each system under consideration, and are fitted to the exponential cumulative distribution function. Through this fitting, we estimate the characteristic time of the Poisson process, which is the mean residence time we aim to determine (see also eq S5). The fitting in question is presented in Figure S7 for both DNA–berubicin complexes. Further details about the statistical analysis described in this paragraph are provided in Section S6 of the Supporting Information. The sum of the squared residuals quantifies the variance between the cumulative distribution function derived from the simulation data and that of the exponential distribution. The position of the global minimum of this function corresponds to the mean residence time; this correspondence is explained in detail in the Supporting Information (Section S6). Figure S8 displays the sum of the squared residuals as a function of the natural residence time for the two complexes under consideration. The positions of the global minimum of the blue and red curves in Figure S8 correspond to the natural residence times for 5′-d­(ACGTAC|GT)-3′–berubicin (1.07 s ± 0.05 s) and 5′-d­(TGT|ACA)-3′–berubicin (0.10 s ± 0.01 s), respectively. It should be noted that in both cases the two-sample Kolmogorov–Smirnov test is satisfied, as indicated by the *p*-values: 0.42 (5′-d­(ACGTAC|GT)-3′–berubicin) and 0.81 (5′-d­(TGT|ACA)-3′–berubicin). The temperature dependence of the rate constants is investigated by applying the well-known Eyring equation (see eq 2), which is widely employed for describing the transition state of complex molecular systems.

2 $$k=\frac{\kappa k_{B}T}{h}\exp\left(-\frac{\Delta G^{‡}}{\mathrm{RT}}\right)$$

where *h* and *k*_B_ are the Planck and Boltzmann constant, respectively, and Δ*G* ^‡^ denotes the activation Gibbs energy. The transmission coefficient κ is set to unity, an approximation entailing that every time the system reaches the transition state, it succeeds to cross it and find itself to the other stable state.^100^ The activation Gibbs energies for the 5′-d­(ACGTAC|GT)-3′–berubicin and 5′-d­(TGT|ACA)-3′–berubicin complexes are 76.2 ± 0.1 and 70.2 ± 0.3 kJ/mol, respectively, values that are close to each other. The related literature interprets these high barriers in terms of significant conformational changes to the anthracyclines, as well as the DNA structures themselves, upon opening/closing of the intercalation site.^29,54,76,95,101^ Figure 9, which concerns the 5′-d­(ACGTAC|GT)-3′ sequence, visualizes the conformational changes. It is noted that similar conformational changes are observed for 5′-d­(TGT|ACA)-3′. Additionally, many of the rate constants reported in the literature are for temperatures different from the one used in our molecular dynamics simulations (310 K). For this purpose, we employ the following equation, derived by applying eq 2 at two distinct temperatures, *Τ*_1_ and *Τ*_2_ on the assumption of similar transmission coefficients at the two temperatures:

3 $$\frac{k_{1}}{k_{2}}=\frac{\tau_{2}}{\tau_{1}}=\frac{{Τ}_{1}}{{Τ}_{2}}\cdot \exp\left[-\frac{\Delta G^{‡}}{R}\left(\frac{1}{T_{1}}-\frac{1}{T_{2}}\right)\right]$$

**Figure 9:**
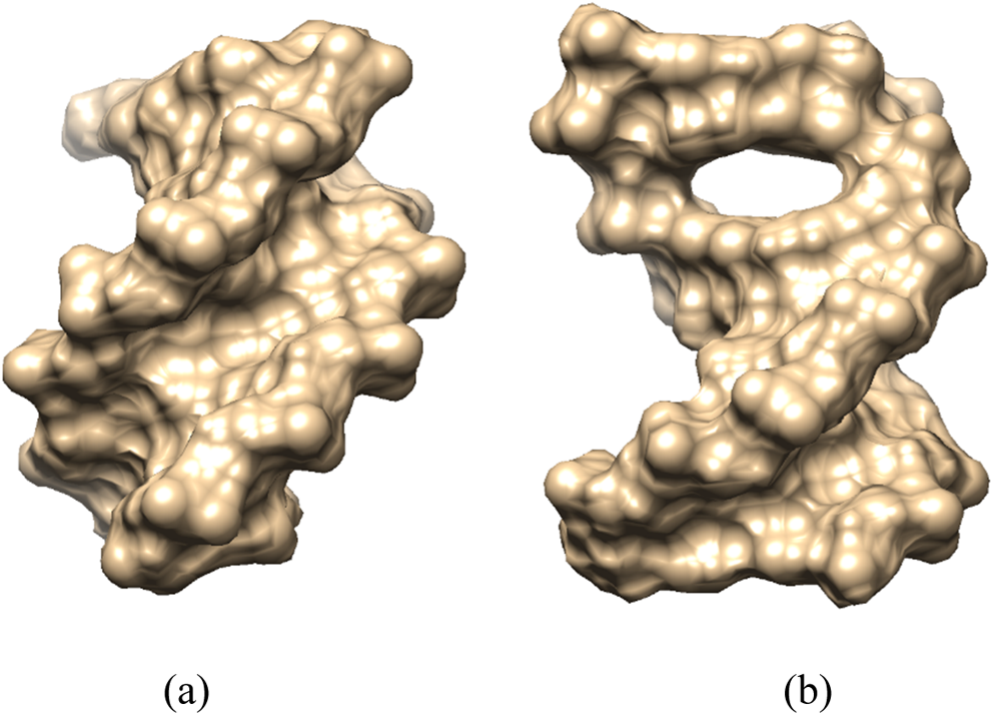
Visualization of 5′-d­(ACGTAC|GT)-3′ when free in water (a) and when berubicin is bound to its intercalation site (b). The oligonucleotides are depicted as molecular surfaces using the CHIMERA software.^104^

Mean residence times at three different temperatures, as computed from our simulations, are gathered in Table 4. For comparison purposes, mean residence times of doxorubicin and daunorubicin, which are considered prototype molecules of the anthracycline class, are summarized in Table 5. These values are obtained from experimental studies on DNA–anthracycline complexes. It is noted that, to the best of our knowledge, there are no published mean residence times for berubicin. The first conclusion from Tables 4 and 5 is that most reported mean residence times fall within the same order of magnitude. In comparison, anthraquinone alone, which binds to intercalation sites, exhibits mean residence times on the order of milliseconds.^102,103^ In contrast, anthracyclines have significantly longer mean residence times due to additional stabilization from interactions with the minor groove of DNA, which reveals their dual binding mode with DNA.

**Table 4:** Mean Residence Times Calculated by WT-MetaD Simulations at Different Temperatures^a^,^b^

system	310 K	300 K	290 K
5′-d(ACGTAC|GT)-3′–berubicin	1.07 ± 0.05	2.96 ± 0.17	8.79 ± 0.51
5′-d(TGT|ACA)-3′–berubicin	0.10 ± 0.01	0.26 ± 0.03	0.70 ± 0.07

a The simulations are conducted at a temperature of 310 K. At the other reported temperatures, the mean residence times are calculated using eq 3.

b The mean residence times are given in seconds.

**Table 5:** Mean Residence Times for Doxorubicin and Daunorubicin from Published Experimental Studies^a^,^b^,^c^

system	310 K	300 K	290 K
DNA–doxorubicin	0.10 ^23^	0.48,^23,75^ 1.25 ^26^	0.28,^98^ 0.29,^24^ 0.54,^23^ 1.82 ^105^
DNA–daunorubicin	0.09,^23^ 0.1 ^27^	0.5,^27^ 0.40,^75^ 0.46,^23^ 1.08 ^26^	0 × 10^24^, 0.12,^98^ 0.35,^106^ 0.70,^23^ 1.03,^99^ 1.47 ^105^

a The mean residence times are obtained from the inverse of the deintercalation rate constant. If there are multiple deintercalation rate constants, then the smallest one is selected, corresponding to the first deintercalation stage, which is the rate-limiting step.^27^

b While some articles report data between 288 and 294 K, we standardized these values to 290 K in this table. Similarly, reported 298–299 K values were approximated as 300 K, whereas 306 K was approximated as 310 K.

c The mean residence times are given in seconds.

The mean residence time of the 5′-d­(ACGTAC|GT)-3′–berubicin complex is higher compared to that of the 5′-d­(TGT|ACA)-3′–berubicin complex; this difference is primarily attributed to the structure and energetics of the intercalation site itself. Regarding the sequence of the intercalation site, 5′-d­(ACGTAC|GT)-3′ has a 5′-d­(C|G)-3′ base-pair step as intercalation site, while 5′-d­(TGT|ACA)-3′ has a 5′-d­(T|A)-3′ base-pair step. The former is characterized by a more asymmetric charge distribution; thus, it forms stronger electrostatic interactions than the latter, when the ligand is a polar molecule,^107,108^ which affects the deintercalation rate.^109^ Another difference between these base pairs lies in the stacking enthalpy required to separate them. According to the literature, the 5′-d­(CG)-3′ base-pair step is more tightly formed than the 5′-d­(TA)-3′ one.^110^ To validate this, we calculated the mean total interaction energy between the consecutive base pairs at both intercalation sites in their unbound states. We found that 5′-d­(ACGTAC|GT)-3′ exhibits a more negative total interaction energy, differing by approximately 7.5 kJ/mol. This causes a larger activation Gibbs energy for intercalation into the 5′-d­(C|G)-3′ base-pair step compared to the 5′-d­(T|A)-3′ step. This implies a slower intercalation rate, *k*_on_, into the 5′-d­(C|G)-3′ base-pair step because the favorable stacking interactions are more difficult to break. This has been observed in several experimental studies concerning DNA–ligand intercalations (including anthracyclines) using synthetic polynucleotides.^26,107,111^ The intercalation rate is related to the deintercalation rate, *k*_off_, through the following equation: $K_{\mathrm{eq}}=\frac{k_{\mathrm{on}}}{k_{\mathrm{off}}}$ , where *K*_eq_ is the intercalation equilibrium constant which is a direct function of Δ*G*_bind_^°^. As demonstrated in Table 3 and discussed in ref 11, the 5′-d­(ACGTAC|GT)-3′–berubicin and 5′-d­(TGT|ACA)-3′–berubicin complexes exhibit Δ*G*_bind_^°^ values within statistical error, which correspond to similar *K*_eq_ values. Thus, a slower intercalation of 5′-d­(ACGTAC|GT)-3′–berubicin compared to 5′-d­(TGT|ACA)-3′–berubicin would lead to a slower deintercalation as well, or equivalently, a longer mean residence time. The difference in the oligonucleotide sequences is reflected in terms of hydrogen bonding, greater shielding effects to the surrounding water molecules and stronger hydrophobic interactions associated with 5′-d­(ACGTAC|GT)-3′; a detailed description of these three factors is provided later in this article.

Next, we proceed to a geometric description of the three states involved in the deintercalation process. The reader is reminded that the second (reshuffling) state is not a stable state, as it does not correspond to a Gibbs energy basin. It is an intermediate metastable state, represented by a wide Gibbs energy plateau between the two stable ones, during which significant conformational changes occur in both DNA and berubicin. The discussion in this paragraph focuses only on the 5′-d­(ACGTAC|GT)-3′–berubicin complex because the 5′-d­(ACGTAC|GT)-3′ oligomer is larger than 5′-d­(TGT|ACA)-3′, making the DNA geometric changes more evident. As seen by the sequence of 5′-d­(ACGTAC|GT)-3′ given in Section 2, the intercalation site is located at the sixth base-pair step. It should be stressed at this point that the terminal base pairs are not taken into account due to fraying effects.^33,112^ The helical parameters define the three-dimensional structure of DNA. For this reason, we investigate the effect of berubicin on the selected DNA sequences in terms of the following two helical parameters: twist and rise, which exhibit the most significant changes upon deintercalation in this study, as well as in the related literature.^29,74,113–115^ In brief, the rise is the perpendicular distance between two successive (nitrogenous) base pairs, whereas the twist is the angle of rotation around the helical axis between two consecutive base pairs.^116^ In addition to the two aforementioned properties, we calculate the base-pair step overlap which is defined as the total overlap surface between the atoms of two successive base pairs.^116^ The 3DNA 2.0 web server^116,117^ is employed to conduct all geometric calculations analyzed and discussed in this paragraph.

Figure 10 shows the three properties in question as a function of the base-pair step; the terminal base pairs are excluded. The labels *unbiased free DNA* and *unbiased intercalated state* refer to systems simulated in ref 11, corresponding to 5′-d­(ACGTAC|GT)-3′ free in water and protonated berubicin bound with 5′-d­(ACGTAC|GT)-3′ in the intercalated state, again in water. The remaining curves in Figure 10 pertain to the systems investigated in this article, specifically focusing on the three states under examination. The base-pair overlap generally takes successive values of 1–2 and 10–11 Å due to continuous purine-pyrimidine repetition in the examined DNA sequence.^118–120^ However, at the intercalation site, the value of the base-pair overlap drops to zero, since there is no overlap. This is due to a synergistic effect of the untwisting and shift of the intercalation site’s base pair toward the major groove, as observed in this article, as well as in other studies.^11,81,91^ As concluded from Figure 10a, in all cases where the intercalation is closed, the property in question exhibits nonzero values at the sixth base pair. It is noteworthy that the base-pair overlap in the reshuffling state (1 Å) falls between those of the intercalated state and the minor groove-bound state.

**Figure 10:**
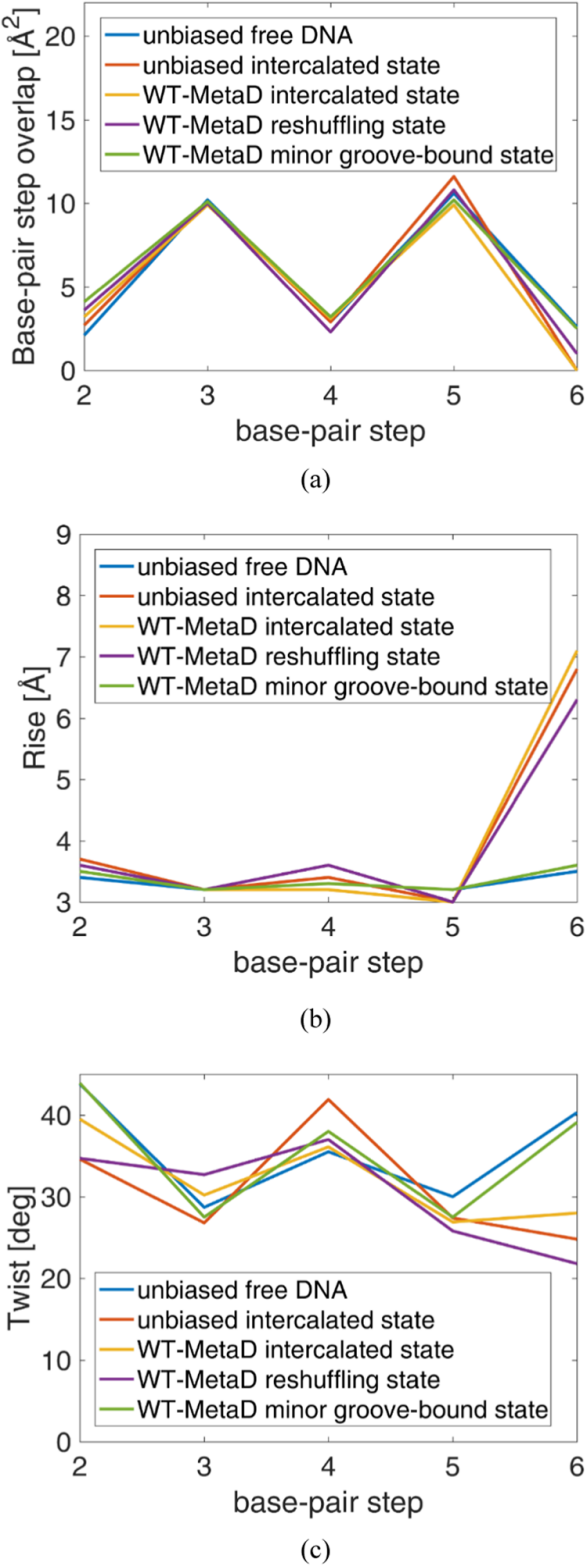
Geometrical properties of 5′-d­(ACGTAC|GT)-3′ as a function of base-pair step during deintercalation, compared to unbound and bound DNA states from unbiased molecular dynamics simulations:^11^ (a) base-pair step overlap, (b) rise and (c) twist.

In Figure 10b, it is observed that at the sixth base pair step, the rise of the unbiased free DNA and of the minor groove-bound state exhibit values (≈3.3 Å) that are in full agreement with those reported in the literature for B-DNA.^116,121,122^ However, the remaining systems manifest a sharp increase in this property at the sixth base pair. Again, as in the case of the base-pair step overlap, the rise of the sixth base pair step in the reshuffling state lies between those of the intercalated state and the minor groove-bound state, indicating the gradual breakdown of the intercalation site. The drop at the fifth base-pair step (2.8 Å), which is below the average value of 3.3 Å, is interpreted as a squeeze caused by the extensive rise at the sixth one. Such geometric changes around the intercalation site of double-stranded DNA sequences have been observed in other computational studies as well.^29,74,113–115^ As for twist, first, it should be mentioned that our values for the free DNA in an aqueous environment are fully consistent with published data for B-DNA.^116,121,122^ Then a small drop of this quantity is observed at the sixth base-pair step in the intercalated state, as expected from the related literature.^29,74,113,114^ Also, it is seen from Figure 10c that the twist of the reshuffling state at the sixth base-pair step demonstrates the lowest value, which is due to the collapse of the interaction site during the transfer of the berubicin from the intercalated to the minor groove-bound state. In the case of twist, we find that the reshuffling state does not exhibit values between the two stable states, a trend that has been observed for the twist,^29,74^ as well as for other helical parameters not examined in this article. Lastly, it should be pointed out that for all properties in Figure 10 the plots corresponding to the *unbiased intercalated state* are similar to those of the *WT-MetaD intercalated state*, confirming that the sampling is adequate and reliable. At the same time, the *unbiased free DNA* plots are similar with the *WT-MetaD minor groove-bound state* ones. The latter result confirms that the minor groove-bound DNA adopts a B-DNA conformation, consistent with discussions above in this section.

Our analysis continues with some properties shedding light on the driving and stabilizing factors of each of the three states under examination. The relevant data for both the 5′-d­(TGT|ACA)-3′–berubicin and 5′-d­(ACGTAC|GT)-3′–berubicin complexes are summarized in Table 6. The solvent accessible surface area (SASA) of berubicin decreases upon complexation in all bound states relative to its value when the drug is free in water. The lowest SASA values are observed in the intercalated state of both complexes, since both the anthraquinone and sugar moieties are buried to a significant extent. As berubicin comes out of the intercalation site, the SASA increases but remains lower than that in its free state. The physical meaning of these changes is that, during the transition from the intercalated state to the minor groove-bound state, the drug becomes more exposed to the surrounding water phase. It should also be noted that the SASA values of berubicin are smaller when bound to the 5′-d­(ACGTAC|GT)-3′ sequence. This finding aligns with the longer-lasting interaction of berubicin with 5′-d­(ACGTAC|GT)-3′ from a kinetic perspective; the enhanced shielding of berubicin from the surrounding water phase provided by 5′-d­(ACGTAC|GT)-3′ kinetically stabilizes the intercalated state. The stronger interactions in the case of the 5′-d­(ACGTAC|GT)-3′–berubicin complex, observed across all states, are also reflected by the higher number of hydrogen bonds formed between the two constituent molecules. The greater number of hydrogen bonds in the minor groove bound-state for both complexes is in full agreement with the stronger hydrogen bond network formed when the drug is located in the minor groove, as also detailed in ref 77. Furthermore, most of the DNA–berubicin hydrogen bonds are formed in the minor groove of DNA.^11^ According to ref 11 and references therein, the stabilization of the DNA–berubicin complexes in the intercalated state is primarily driven by nonelectrostatic interactions, such as stacking interactions, whereas the electrostatic interactions, including hydrogen bonding, become important in the minor groove-bound state.^77,115^ Another notable observation regarding the hydrogen bonding is the abrupt drop in the number of hydrogen bonds formed between berubicin and the two DNA structures in the reshuffling state, attributed to a sudden influx of water molecules resulting from the gradual exit of berubicin from the intercalated state. This supports the idea that the intramolecular and intermolecular hydrogen bonds in the receptor–ligand complex kinetically stabilize the complex by keeping the water molecules away from the binding pocket.^11,62,109,123^ The just-described hydration of the binding pocket participates in the unbinding in many cases of noncovalent complexes, such as protein–drug complexes.^28,53,56,62,109,123,124^ Furthermore, in a number of published articles, it is detailed that the high activation energies in noncovalent unbinding are attributed partly to the disruption of the above-mentioned shielding and the resulting entrance of surrounding water molecules into the binding pocket.^53,109,123^

**Table 6:** Various Properties Concerning the Three Stages of the Deintercalation Process^a^

property	5′-d(ACGTAC|GT)-3′	5′-d(TGT|ACA)-3′
SASA of berubicin (nm^2^)	9.090 ± 0.002 (free)	9.090 ± 0.002 (free)
	3.91 ± 0.05	4.55 ± 0.06
	5.08 ± 0.26	5.41 ± 0.16
	5.76 ± 0.11	6.38 ± 0.13
berubicin–DNA hydrogen bonds	- (free)	- (free)
	1.55 ± 0.11	0.75 ± 0.06
	0.48 ± 0.14	0.30 ± 0.11
	1.80 ± 0.16	1.48 ± 0.47
Extended interaction site–water hydrogen bonds	54.42 ± 0.37 (free)	55.00 ± 0.05 (free)
	51.53 ± 0.25	53.55 ± 0.32
	54.43 ± 0.46	54.52 ± 0.33
	51.13 ± 0.25	52.71 ± 0.32
Water molecules within 5.0 Å from the COM of the extended interaction site	167.8 ± 0.2 (free)	171.7 ± 0.1 (free)
	164.2 ± 0.4	168.8 ± 0.4
	166.6 ± 1.5	172.1 ± 1.2
	153.2 ± 0.6	157.8 ± 1.1
berubicin–DNA van der Waals interactions (kJ/mol)	- (free)	- (free)
	–250.0 ± 2.5	–215.4 ± 2.3
	–180.6 ± 14.2	–162.9 ± 6.9
	–139.8 ± 7.5	–98.3 ± 6.0
berubicin–DNA electrostatic interactions (kJ/mol)	- (free)	- (free)
	–94.9 ± 5.3	–72.6 ± 5.2
	–54.7 ± 10.6	–38.6 ± 11.6
	–94.8 ± 4.7	–114.5 ± 23.4
Δ*G*_hpb_ (kJ/mol)	- (free)	- (free)
	–48.4 ± 2.3	–41.9 ± 1.4
	–38.1 ± 5.1	–38.7 ± 2.8
	–37.6 ± 3.3	–27.8 ± 3.2

a The first line in each block refers to the free state in water, whether it is berubicin or the oligonucleotide DNA. The second, third, and fourth lines in each block correspond to the intercalated, reshuffling, and minor groove-bound states, respectively.

As stated in Section S4, there are three base pairs that significantly interact with berubicin, i.e., the two base pairs of the intercalation site and the next one toward the minor groove. The extended interaction sites are 5′-d­(AC|G)-3′ and 5′-d­(T|AC)-3′ for 5′-d­(ACGTAC|GT)-3′ and 5′-d­(TGT|ACA)-3′, respectively. Two relevant properties, presented in Table 6, are the number of hydrogen bonds between the extended interaction sites and water molecules, and the count of water molecules within 5.0 Å of the COM of the extended interaction site. Both these properties provide insight into the hydration of the extended interaction site during the deintercalation stages. It is observed that in both cases, there are more water molecules in that region in the unbound DNA than in the bound states. Thus, the drug expels water molecules when bound to DNA, as expected in general for DNA–drug intercalations.^59,125–127^ Table 6 indicates the aforementioned attraction of water molecules during the first deintercalation stage (from the intercalated to the reshuffling state): water molecules invade the extended interaction site, forming new hydrogen bonds with both the DNA and berubicin, thereby breaking the stabilizing forces of the intercalated state, and thus contributing to the extraction of berubicin from the intercalation site. When berubicin and DNA attain their new conformation by passing into the minor groove-bound state, some water molecules are again expelled by berubicin. Comparing the two oligonucleotides, we observe that water molecules are more effectively expelled from the 5′-d­(ACGTAC|GT)-3′ structure than from the 5′-d­(TGT|ACA)-3′ structure. This bolsters our statement that the shielding effect is stronger in 5′-d­(ACGTAC|GT)-3′, contributing to the slower deintercalation from it, and that water molecules play an important role during the deintercalation process. The water-mediated unbinding, as detailed herein, is also critically examined in numerous *in silico* studies concerning protein–ligand complexes.^53,56,61^

The next two properties presented in Table 6 are the van der Waals and electrostatic contributions to the potential energy between DNA and berubicin in each of the three states. In both complexes, the van der Waals interactions prevail over the electrostatic interactions when DNA and berubicin are in the intercalated state, whereas the difference between these two types of interactions becomes much smaller in the minor groove-bound state. Especially in the case of the 5′-d­(TGT|ACA)-3′–berubicin in the minor groove-bound state, the electrostatic contributions become dominant. This is in accordance with the literature, as we mentioned above, stating that the intercalation of anthracyclines into DNA is driven by nonelectrostatic and hydrophobic interactions, while electrostatic interactions become more significant in the minor groove bound state.^59,77^

The next property is the hydrophobic contribution to the binding Gibbs energy, Δ*G*_hpb_, which is estimated using the next equations:^128^

4 $$\Delta G_{\mathrm{hpb}}\left[\mathrm{cal}/\mathrm{mol}\right]=80\Delta C_{p}\left[\mathrm{cal}/\mathrm{mol}/K\right]$$

5 $$\Delta C_{p}\left[\mathrm{cal}/\mathrm{mol}/K\right]=0.382\cdot \Delta \mathrm{SAS}A_{\mathrm{np}}\left[{Å}^{2}\right]-0.121\cdot \Delta \mathrm{SAS}A_{p}\left[{Å}^{2}\right]$$

Here Δ*C*_p_ stands for the change in the heat capacity at constant pressure of the system from the unbound to a bound state. ΔSASA_np_ and ΔSASA_p_, which refer to both DNA and berubicin, denote the changes in the nonpolar and polar contributions of the SASA, respectively. All carbon and phosphorus atoms, along with the hydrogen atoms covalently bonded to carbons, are considered nonpolar. The remaining atoms are treated as polar.^128^ The Δ*G*_hpb_ values are shown in Table 6, whereas Δ*C*_p_, ΔSASA_np_, and ΔSASA_p_ are shown in Table S1 in the Supporting Information. First, it is pointed out that the Δ*G*_hpb_ and Δ*C*_p_ values are in agreement with experimental and computational studies concerning other anthracyclines.^128,129^ In all three states, both ΔG_hpb_ and Δ*C*_p_ exhibit large negative values, which highlight the significance of hydrophobic interactions.^127,130,131^ As discussed above, the hydrophobic interactions in the intercalated state are more significant, a finding that is also supported by the reported values of Δ*G*_hpb_ in Table 6. Furthermore, a comparison between the 5′-d­(ACGTAC|GT)-3′–berubicin and 5′-d­(TGT|ACA)-3′–berubicin complexes, in terms of Δ*G*_hpb_, indicates that 5′-d­(ACGTAC|GT)-3′ displays stronger hydrophobic interactions with berubicin, which is ascribed to the more extensive burial of berubicin, thereby providing more efficient shielding from water molecules when bound in the intercalation site of that DNA oligonucleotide. These factors indicate that the intercalation site sequence of 5′-d­(ACGTAC|GT)-3′, 5′-d­(C|G)-3′, results in a greater steric complementarity with the structure of berubicin than the intercalation site sequence of 5′-d­(TGT|ACA)-3′, 5′-d­(T|A)-3′. It is noted that steric complementarity is a significant stabilizing factor in most receptor–ligand complexes.^132^ Despite the aforementioned difference, the two DNA–berubicin complexes exhibit standard binding Gibbs energies within statistical error. The primary reason is that the stronger hydrophobic interactions between 5′-d­(ACGTAC|GT)-3′ and berubicin are counterbalanced by the higher free energy penalty for desolvation, compared to the 5′-d­(TGT|ACA)-3′–berubicin complex.^11^ However, this difference in steric complementarity affects the kinetics, making deintercalation from 5′-d­(ACGTAC|GT)-3′ slower than deintercalation from 5′-d­(TGT|ACA)-3′. Moreover, the higher desolvation free energy penalty for 5′-d­(ACGTAC|GT)-3′ delays intercalation rate, which has an impact on the deintercalation rate, as discussed previously in this section. The impacts of steric and desolvation effects on the binding and unbinding kinetics have been extensively discussed in the literature.^133,134^

Another significant effect taking place in the intercalation and minor-groove binding processes of positively charged drugs to DNA is the cation release from the region near their binding sites.^27,135–137^ In general, the salt concentration plays an important role in thermodynamics, and the binding affinity depends on this concentration.^127,135–137^ When DNA is free in water, it is surrounded by many cations, due to its high negative charge.^127^ The analysis is carried out in terms of the average number of sodium ions within 5.0 Å from the COM of the extended interaction site in each of the three states, as well as in the unbound state of the DNA sequences; these results are summarized in Table 7. It is deduced that approximately one sodium ion is released upon intercalation from both oligonucleotides. In the related literature, the stoichiometry of cation release is described by the *-SK* parameter which is defined as -*SK* = ∂ln *K*_eq_/∂ln­[*MX*], where [MX] is the salt concentration.^127,138^ The release of one cation calculated in this study agrees with prior experimental and computational studies on positively charged anthracycline intercalations in which −*SK* falls between 0.90 and 1.34.^83,101,113,126,129,138,139^ The release in question is associated with two factors. The positively charged drug electrostatically repels the cations. This can be interpreted as an ion exchange process, where the number of released ions should be commensurate with the net charge of the drug.^127^ The second factor involves local DNA extension due to intercalation.^102,139^ As seen in Figure 10b, rise is significantly increased upon intercalation. This results in a drop in absolute value of the DNA charge density,^102,139^ because its length is increased under constant total charge. DNA is a polyanion, and therefore, this charge density decrease reduces its ability to attract cations. In Table 7, we see that there are fewer sodium ions near the interaction site in the intercalated state than in the minor groove-bound state in both oligonucleotides. This is attributed to the fact that in the intercalated state, both the aforementioned cation-releasing factors are present, whereas in the minor groove-bound state, only the first factor is present. Another reason for the reduced cation repulsion in the minor groove-bound state is the location of the protonated amine group of berubicin. In Figures 6 and S3, we observe that this group is close to the phosphate groups in the intercalated state, whereas it is exposed to the surrounding water phase in the minor groove-bound state. This is important, because the phosphate groups carry the negative charge of DNA, and thus, cations primarily surround those groups.^91^ However, there are still 0.3 to 0.7 sodium ions displaced in the minor groove-bound state. As a final point, the presented analysis supports the idea that the electrostatic replacement via the cation-exchange process, driven by the net charge of the drug, plays a more significant role in ion release than the reduction in DNA charge density.

**Table 7:** Number of Sodium Ions within 5.0 Å of the Extended Interaction Site^a^

property	5′-d(ACGTAC|GT)-3′	5′-d(TGT|ACA)-3′
Sodium ions within 5.0 Å from the COM of the extended interaction site	3.74 ± 0.04	2.80 ± 0.03
	2.72 ± 0.06	2.03 ± 0.06
	2.73 ± 0.35	2.50 ± 0.07
	3.00 ± 0.11	2.48 ± 0.05

a The first row in each block refers to the DNA sequences when free in water. The second, third, and fourth rows in each block correspond to the intercalated, reshuffling, and minor groove-bound states, respectively.

## 4 Conclusions

The WT-MetaD simulations reported herein reveal that the deintercalation of berubicin from double-stranded DNA follows a three-step mechanism, progressing from the intercalated state to the minor groove-bound state via an intermediate reshuffling state, during which molecular geometries experience continuous structural rearrangements, and finally to the fully unbound state. The first stage of the mechanism (intercalated to reshuffling state) is the rate-limiting step and the estimated deintercalation times of berubicin are similar, or even larger, compared to anthracyclines in widespread clinical use, such as doxorubicin and daunorubicin. It is pointed out that the three-step mechanism identified is in agreement with published *in silico* and experimental studies concerning both the DNA–anthracycline deintercalation and intercalation processes.^27,29^ Indeed, the intercalation process of anthracyclines into double-stranded DNA follows a three-step mechanism in which an external complex is initially formed.^27,29^ This external complex has been identified to be a minor groove-bound complex, similar to those described in previous studies.^29,74,76,140^ From a medicinal chemistry point of view, the slow transition to the reshuffling state upon deintercalation make the DNA–anthracycline complexes last longer, providing them a significant advantage over other simple DNA intercalators.^27^

It should be mentioned that berubicin, as demonstrated in this study and in ref 11, is a strong DNA intercalator from a thermodynamic point of view. Its binding is tighter than that of doxorubicin and daunorubicin, owing to additional stabilization provided by the interactions between the DNA minor groove and the larger sugar moiety of berubicin due to the presence of the additional methoxy aromatic ring attached on it. The aforementioned stabilization also influences positively the kinetics, as evidenced by the estimated deintercalation times. In addition to deintercalation kinetics, we also investigate the factors that stabilize each state and, consequently, slow down the overall unbinding process, thus providing insights for an efficient drug design protocol.^141^ A significant dependence of the deintercalation time on the intercalation site sequence is found. This dependence is attributed to the shielding of water molecules, the hydrophobic and electrostatic interaction, as well as the cation release.

This study may also serve as a starting point for developing new anthracycline-class drugs by introducing chemical modifications that stabilize the specific states of the deintercalation mechanism, and especially by prolonging the lifetime of the intercalated state. Berubicin, as seen in Figure 1, may be divided into three regions: anthraquinone, anchor and sugar moiety. The anthraquinone intercalates between two successive DNA base pairs, while the anchor and sugar moiety both form hydrogen bonds with DNA; the sugar moeity additionally engages the minor groove through favorable electrostatic interactions, facilitated by its positive charge. In ref 11, it is shown that berubicin forms more stable DNA intercalation complexes than doxorubicin and daunorubicin. This is attributed to the enhancement of the hydrophobic character of the molecule by inserting the extra aromatic ring in the sugar moiety. We could further stabilize the intercalation complex by enhancing the hydrogen bonding character of the anthracycline. The net positive charge of anthracyclines is vital for intercalation, as demonstrated in this work and ref 11 . This stabilization could be enhanced by selecting suitable side groups that increase the molecule’s polarity or by changing the charged group. This should be done with caution; otherwise, the hydrophilicity will increase significantly and intercalation will no longer be favorable. From a kinetic perspective, repelling water molecules and cations from the vicinity of the binding site prolongs the bound state. A practical strategy to achieve this is the incorporation of hydrophobic groups into the berubicin structure without the intercalation being hindered stereochemically. All the aforementioned modifications should be implemented carefully to avoid undesired effects in drug discovery, such as molecular obesity.^142^

## Supplementary Material



## Notes

Graphical depictions and data concerning the 5′-d­(TGT|ACA)-3′–berubicin complex (Section S1), a table of SASA and thermal capacity changes (continuation of Table 6; Section S2), the details about WT-MetaD and the methods in use (Section S3), the derivation of eq 1 (Section S4), a discussion of error analysis and convergence tests for WT-MetaD simulations (Section S5), details on extracting mean residence times and the associated error analysis (Section S6), justification of the used WT-MetaD parameters (Section S7) (PDF)
