# Supplementary material for: Thermodynamics and Kinetics of the Deintercalation of a Novel Anthracycline from Double-Stranded Oligonucleotide DNA
Source: J Phys Chem B. 2025 Aug 12;129(33):8335–50. doi: 10.1021/acs.jpcb.5c03021 (PMC12376102; doi:10.1021/acs.jpcb.5c03021)
Supplement: Supplementary file 1 [file jp5c03021_si_001.pdf]

SUPPORTING INFORMATION

Thermodynamics and Kinetics of the Deintercalation of a Novel Anthracycline from Double-Stranded Oligonucleotide DNA

Georgios Mikaelian,[(a)] *Haralambos Sarimveis,*[(a)] *Doros N. Theodorou*[(a,b)] *and Grigorios Megariotis*[(a,c)(*)]

[(a)] School of Chemical Engineering, National Technical University of Athens (NTUA), 9 Heroon Polytechniou Street, Zografou Campus, Athens, GR, 15780, Greece

[(b)] Academy of Athens, 28 Panepistimiou Street, GR-10679 Athens, Greece

[(c)] School of Engineering, Department of Mineral Resources Engineering, University of Western Macedonia, 50100, Kozani, Greece.

[(*)]gregm@mail.ntua.gr

S1. GRAPHICAL DEPICTIONS AND DATA CONCERNING THE 5′-d(TGT|ACA)-3′ – BERUBICIN COMPLEX

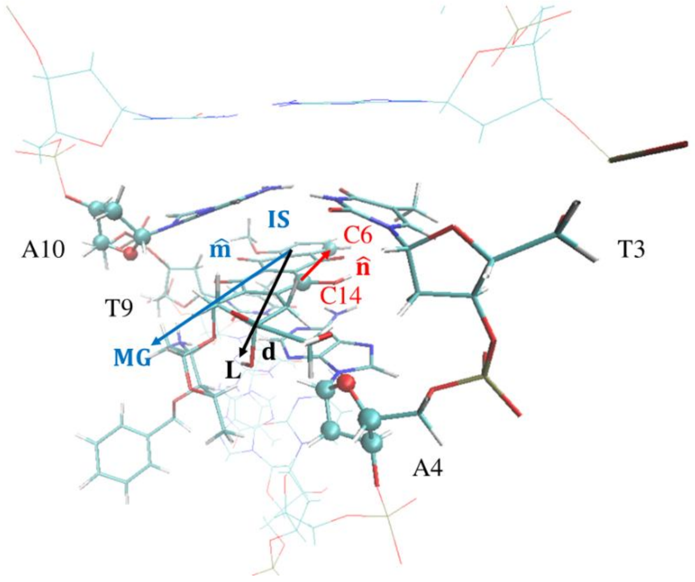

Figure S1. Schematic representation of the points and vectors employed for the definition of the two CVs for the 5′-d(TGT|ACA)-3′ – berubicin complex.

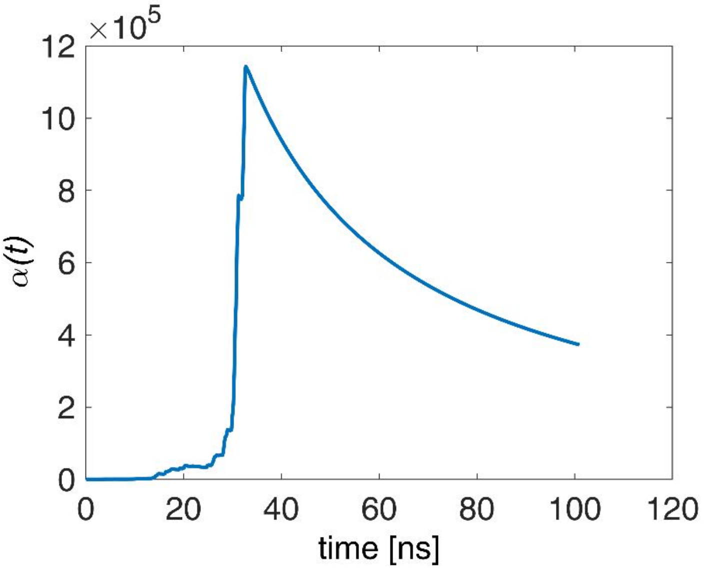

Figure S2. Acceleration factor as a function of simulation time for a single simulation of 5′-d(TGT|ACA)-3′ – berubicin complex.

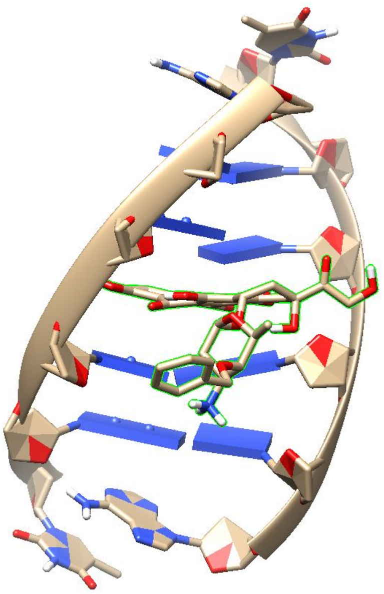

(a)

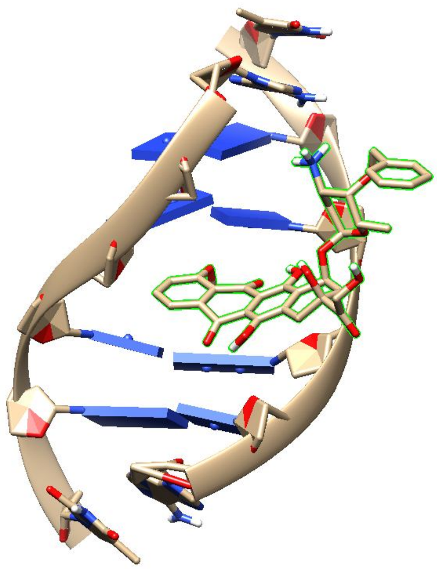

(b)

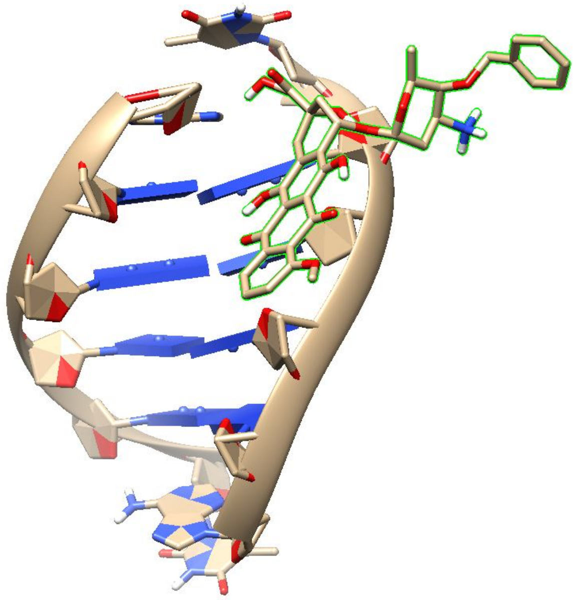

(c)

Figure S3. Representative configurations of the 5′-d(TGT|ACA)-3′ – berubicin complex during the transition from the intercalated state to the minor groove-bound state. (a) intercalated state, (b) reshuffling state and (c) minor groove-bound state.

S2. CHANGES IN SOLVENT ACCESSIBLE SURFACE AREA AND HEAT CAPACITY UPON DNA – BERUBICIN BINDING

Table S1. Terms appearing in eqs 4, 5[a]

Property	5′-d(ACGTAC|GT)-3′	5′-d(TGT|ACA)-3′
$\Delta SASA_{np}$ (nm^2)	-4.5 ± 0.2	-4.0 ± 0.1
	-3.5 ± 0.4	-3.4 ± 0.2
	-3.6 ± 0.3	-2.8 ± 0.2
$\Delta SASA_{p}$ (nm^2)	-2.4 ± 0.1	-2.2 ± 0.1
	-1.7 ± 0.1	-1.3 ± 0.2
	-2.1 ± 0.2	-2.0 ± 0.2
ΔC_{p} (cal/mol/K)	-144.5 ± 7.0	-125.2 ± 4.1
	-113.9 ± 15.2	-115.7 ± 8.3
	-112.4 ± 10.0	-83.1 ± 9.6

[a] The first, second and third lines in each block correspond to the intercalated, reshuffling and minor groove-bound states, respectively.

S3. DETAILS ABOUT WELL-TEMPERED METADYNAMICS AND THE COMPUTATIONAL PROTOCOLS IN USE

WT-MetaD, the method of our choice, requires the identification of a proper set of CVs that accurately represent the rare events under consideration. After the selection of the CVs, positive history-dependent potential energy terms are added periodically; these terms are multivariate Gaussian functions of the CVs, centered on the running CV values, which prevent the system from visiting previously sampled points in CV space. The total added bias potential at the current time t, $V_{\mathrm{b}}(CV_1(\mathbf{r}), \ldots, CV_{\mathrm{N}}(\mathbf{r}), t)$, is given by the following equation:

$$V_b(CV_1(\mathbf{r}),\dots,CV_N(\mathbf{r}),t+\Delta t) = \sum_{t_k=\Delta t,2\Delta t,\dots}^{t} \exp\left[-\frac{V_b(CV_1(\mathbf{r}(t_k)),\dots,CV_N(\mathbf{r}(t_k)),t_k)}{R\Delta T}\right] \cdot$$

$$h_0 \cdot \exp\left\{-\sum_{i=1}^{N} \frac{[CV_i(\mathbf{r}) - CV_i(\mathbf{r}(t_k))]^2}{2\sigma_i^2}\right\} \qquad \text{(S1)}$$

$CV_1(\mathbf{r}),\dots,CV_N(\mathbf{r})$ stand for the CVs that in general are functions of the position vectors of all the interaction sites, $\mathbf{r}$, and are updated every Δt. h_0 is the initial Gaussian height, R the ideal gas constant and ΔT a factor that controls the decreasing rate of the added biases, and has temperature units. The term $h_0 \cdot \exp\left\{-\sum_{i=1}^{N} \frac{[CV_i(\mathbf{r}) - CV_i(\mathbf{r}(t_k))]^2}{2\sigma_i^2}\right\}$ describes the traditional metadynamics Gaussian bias potential[65] with h_0, σ_i and N being the height, the standard deviation for the i-th CV, and the dimensionality of the CV space, respectively. Regarding the term $\exp\left[-\frac{V_b(CV_1(\mathbf{r}(t_k)),\dots,CV_N(\mathbf{r}(t_k)),t_k)}{R\Delta T}\right]$, it is the WT extension in which the heights of the biases decrease exponentially with the total bias potential applied, driving the system into convergence.[66]

Once the WT-MetaD has been applied, the Gibbs energy surface, $G(CV_1,\dots,CV_N,t)$, is extracted from the total bias according to the equation:[S1]

$$G(CV_1,\dots,CV_N,t) = -\frac{T+\Delta T}{\Delta T} \cdot V_b(CV_1,\dots,CV_N,t) + c(t) \qquad \text{(S2)}$$

$c(t)$ is a time-dependent offset bias. At convergence, $G(CV_1,\dots,CV_N,t)$ becomes time-invariant, reducing to $G(CV_1,\dots,CV_N)$. To properly account for the effects of the added biases on the Gibbs energy surface, the reweighting procedure described by Tiwary and Parrinello is employed.[S1]

WT-MetaD is used for the calculation of specific thermodynamic properties, as well as to estimate the rates of rare event transitions and especially the mean residence times in stable Gibbs surface basins. The latter is done via an algorithm developed by

Tiwary et al.[67] At this point, it should be noted that the transition state corresponds to a saddle point on the Gibbs energy surface, situated between two basins. The system remains in this state for a short period on the order of a few picoseconds. It should not be confused with the reshuffling state, also known as the partially intercalated state, a state introduced by Chaires et al.[27] to describe an intermediate —and metastable— state between the intercalated and the minor groove-bound state. In the reshuffling state, the molecular geometries of DNA and berubicin undergo continuous adjustments over a period of tens of nanoseconds in a WT-MetaD simulation. Provided that the CVs are able to distinguish between two stable states and the time spent in the transition state between the two basins is significantly shorter than the residence times in the basins, transition state theory is employed to extract the acceleration factor, $\alpha(t)$, and relate it with the unbiased mean residence time via the next set of equations:[67]

$$\alpha(t) = \langle \exp[\beta V_{\mathrm{b}}(CV_1, \dots, CV_{\mathrm{N}}, t)] \rangle_{(\mathrm{b})} \tag{S3}$$

$$\tau = \alpha(t) \cdot \tau_{\mathrm{meta}} \tag{S4}$$

where $\beta = \frac{1}{RT}$, τ_{meta} is the residence time extracted from the WT-MetaD run and τ is the unbiased or natural mean residence time (if no bias were applied). The average $\langle \dots \rangle_{(\mathrm{b})}$ in eq S3 is calculated while the system is found in the basin, and from this average we estimate the mean residence time, τ. As stated in ref 67, a steep kink in $\alpha(t)$ indicates a transition between the two aforementioned basins. Rare events are stochastic processes, and therefore one should carry out multiple independent WT-MetaD runs in order to approach the mean residence time. Salvalaglio et al.[68] consider rare events as Poisson processes since each event is independent of the others and the rare event hypothesis is valid.[68] The exponential distribution models the *waiting time*

until the first event occurs in a Poisson process. Therefore, the mean residence time is estimated from the simulations by a fitting procedure to the cumulative distribution function of the exponential distribution. The probability of observing at least one transition within a time, t_{pass}, in a simulation is given by the following equation:

$$P_{\nu\geq 1}(t_{\text{pass}}) = 1 - \exp\left(-\frac{t_{\text{pass}}}{\langle\tau\rangle}\right) \tag{S5}$$

The mean residence time of the transition, $\langle\tau\rangle$, is computed by the abovementioned fitting procedure. The advantage of this approach is that we can quantitatively test the validity of the above assumptions by conducting the two-sample Kolmogorov-Smirnov test and checking whether the p-value is greater than the considered significance level (0.05).[67,S2,S3] Both the fitting to a Poisson distribution and the Kolmogorov-Smirnov tests are conducted via an in-house computational code. A detailed discussion of the accuracy of the model, as well as of the related error analysis, is found Section S6.

S4. DERIVATION OF THE STANDARD GIBBS ENERGY DIFFERENCE FOR TRANSFER FROM THE UNBOUND STATE TO A GIBBS ENERGY BASIN

The Gibbs energy surface, $G(CV_1, CV_2)$, represents a PMF expressed as a function of the important degrees of freedom of the problem. The factor $\exp\left[-\frac{G(CV_1,CV_2)}{RT}\right]$ is proportional to the probability of finding the system in the range between (CV_1, CV_2) and $(CV_1 + \mathrm{d}CV_1, CV_2 + \mathrm{d}CV_2)$. Hence, this quantity is directly related to the partition function of the system, expressed as a function of the selected CVs. The molar Gibbs

energy change between two different regions in the CV space is given by the following equation:[15,S4]

$$\Delta G_{\mathrm{PMF}} = -RT \cdot \ln\left\{\frac{\iint_{(\mathrm{state}_1)} \exp\left[-\frac{G(CV_1,CV_2)}{RT}\right] \mathrm{d}CV_1 \mathrm{d}CV_2}{\iint_{(\mathrm{state}_2)} \exp\left[-\frac{G(CV_1,CV_2)}{RT}\right] \mathrm{d}CV_1 \mathrm{d}CV_2}\right\} \tag{S6}$$

where (state_1) and (state_2) are the two states of interest and ΔG_{PMF} represents the Gibbs energy difference for transferring the system from (state_2) to (state_1). In the framework of our analysis, (state_2) represents the unbound state, hereafter abbreviated as (ub), and (state_1) corresponds to a Gibbs energy surface basin, (b). ΔG_{PMF} is not the standard Gibbs energy difference for the transfer process under discussion, $\Delta G^{\circ}_{\mathrm{bind}}$; an additional term is required to account for the standard states invoked in the definition of $\Delta G^{\circ}_{\mathrm{bind}}$. The molecules composing the systems under investigation are free to explore the entire volume of the simulation box in the unbound state, V_{sim}, which generally corresponds to concentrations different from the standard concentration of 1 M. The next equation gives the correction term that should be added to eq S6:[12,S5]

$$\Delta G_{\mathrm{conc}} = -RT \cdot \ln\left(\frac{C_{\mathrm{bound}}}{C_{\mathrm{site}} C_{\mathrm{drug}}} \cdot \frac{C^{\circ}_{\mathrm{site}} C^{\circ}_{\mathrm{drug}}}{C^{\circ}_{\mathrm{bound}}}\right) = -RT \cdot \ln\left(\frac{C_{\mathrm{bound}} C^{\circ}}{C_{\mathrm{site}} C_{\mathrm{drug}}}\right) \tag{S7}$$

, where C_{bound} is the concentration of the complex in the bound state, C_{drug} is the concentration of the drug in the unbound state and C_{site} is the concentration of the DNA binding sites.[74,113] In the simulations reported in the main text, there is only one drug molecule that binds to a single binding site in each DNA oligonucleotide; consequently, $C_{\mathrm{bound}} = C_{\mathrm{drug}}$. Then, the standard binding Gibbs energy is provided by the equation below:

$$\Delta G^{\circ}_{\mathrm{bind}} = -RT \cdot \ln\left\{\frac{\iint_{(\mathrm{b})} \exp\left[-\frac{G(X,\theta)}{RT}\right] \mathrm{d}X \mathrm{d}\theta}{\iint_{(\mathrm{ub})} \exp\left[-\frac{G(X,\theta)}{RT}\right] \mathrm{d}X \mathrm{d}\theta}\right\} - RT \cdot \ln\left(\frac{C^{\circ}}{C_{\mathrm{site}}}\right) \tag{S8}$$

where we have replaced the arbitrary CV_1, CV_2, (state_1) and (state_2) with the ones used in this study, namely X, θ, (b) and (ub), respectively. C_{site} equals $C_{\text{DNA}} \cdot \frac{n_{\text{tot}}}{n_{\text{bp}}}$, where n_{bp} is the number of base pairs constituting the binding pocket for the drug and n_{tot} is the total number of base pairs in the oligonucleotide. n_{bp} equals 3 for daunorubicin,[82] a molecule similar to berubicin, and we use this value in our calculations. 5′-d(ACGTAC|GT)-3′ is an octamer (n_{tot} = 8) and 5′-d(TGT|ACA)-3′ is a hexamer (n_{tot} = 6), so C_{site} is $\frac{8}{3}C_{\text{DNA}}$ and $\frac{6}{3}C_{\text{DNA}}$, respectively. The ratio $\frac{C^{\circ}}{C_{\text{DNA}}}$ is equal to $\frac{V_{\text{sim}}}{V^{\circ}}$, where V° = 1.66 nm^3 is the standard volume. Equation S8 can be viewed as a two-leg thermodynamic cycle: first, the drug is transferred from the binding pocket in water to the unbound state in water at an arbitrary concentration, second, it is transferred to the unbound state in water at the standard concentration. Therefore, the left-hand side of eq S8 corresponds to the standard binding Gibbs energy. This is extensively discussed in ref S5.

S5. DETAILS ABOUT THE ANALYSIS AND RELIABILITY OF THE GIBBS ENERGY CALCULATIONS

In order to validate both the energetics and the intermediate stages of deintercalation, we use multiple WT-MetaD runs for the calculation of Gibbs energy surface. This strategy provides a convenient way to estimate the numerical error accompanied with the Gibbs energy calculation.[47,53,54,58,59] The error at each point of the Gibbs energy surface in CV space is calculated as the standard error from the multiple runs and is provided in Figure S4. It is important to be sure that the selected CVs have adequately explored the crucial regions of CV space relevant to the deintercalation process. In WT-MetaD simulations, the heights of the added Gaussian bias potentials should drop

towards zero as time elapses.[66] This trend is apparent in Figure S5, in which the time evolution of an indicative bias height is depicted. Whether this leads to Gibbs energy surface convergence is checked via the one-dimensional PMF that is discussed in the main article. As an indication, the PMF for 5′-d(ACGTAC|GT)-3′ – berubicin is depicted at various time points along a single WT-MetaD run in Figure S6. The last six curves are very similar at X values higher than 0.5 Å.

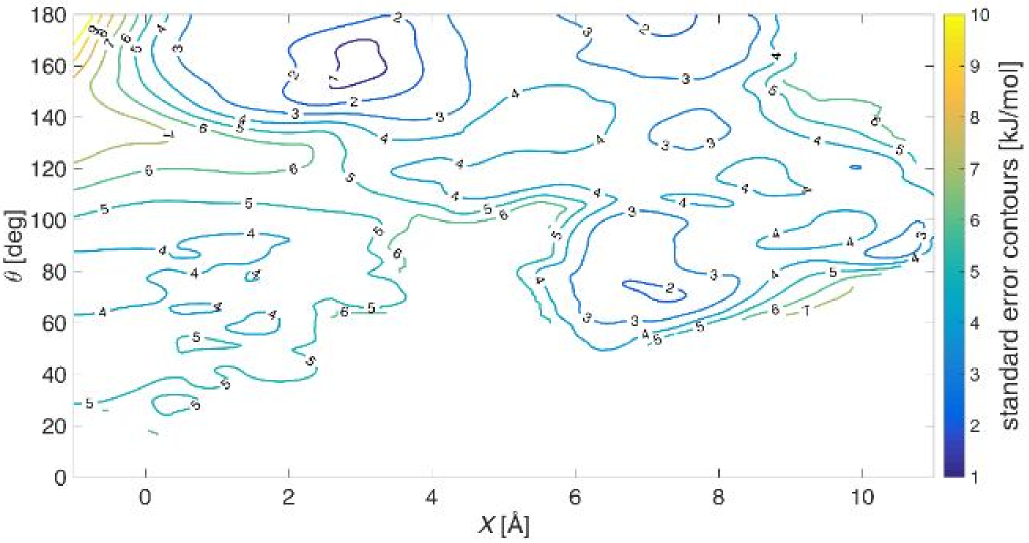

(a)

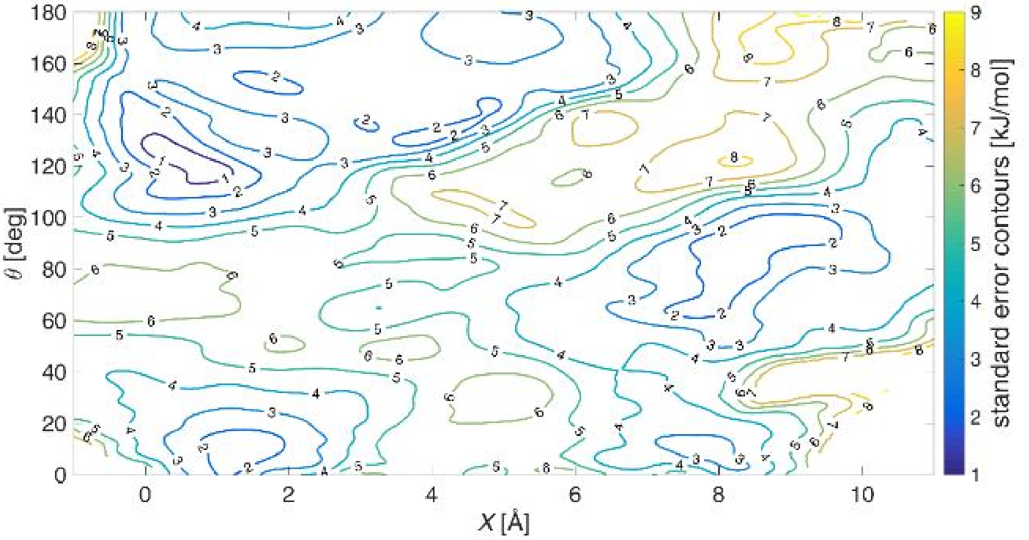

(b)

Figure S4. Contours of Gibbs energy standard error over the CV space for (a) 5′-d(ACGTAC|GT)-3′ – berubicin and (b) 5′-d(TGT|ACA)-3′ – berubicin.

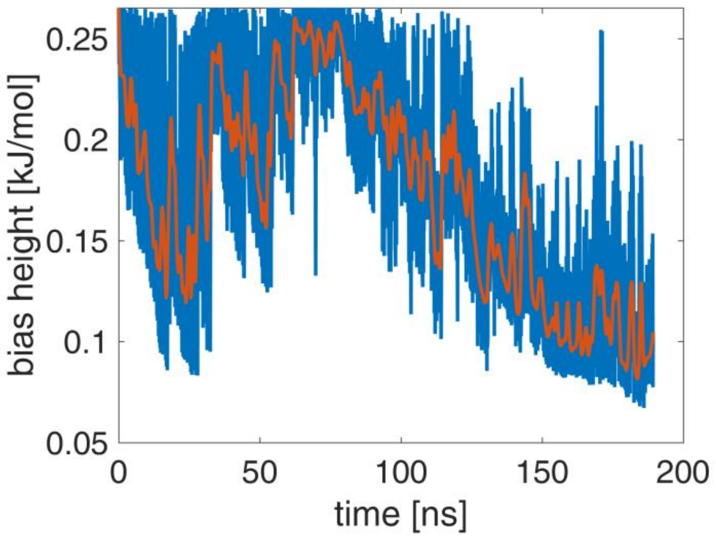

Figure S5. Bias height of the added Gaussians as a function of simulation time along a WT-MetaD run. The blue plot represents the original function, while the orange plot shows the same graph after applying a moving average filter.

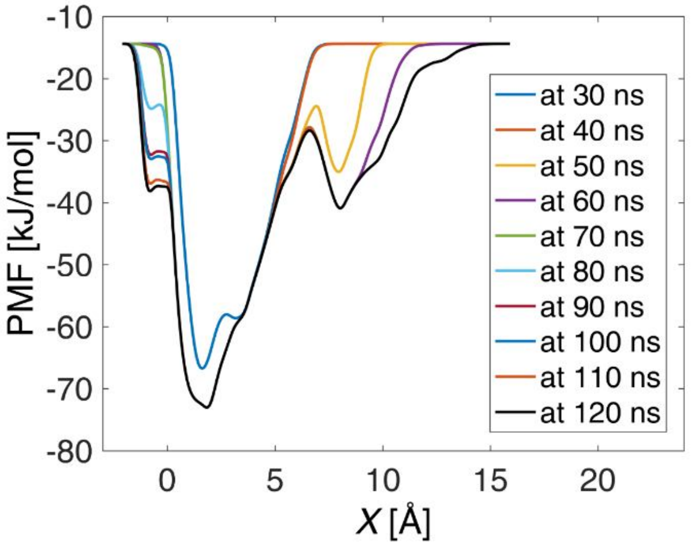

Figure S6. Indicative one-dimensional PMF for 5′-d(ACGTAC|GT)-3′ – berubicin, computed at different times along a single WT-MetaD run.

S6. DETAILS ABOUT THE ANALYSIS AND RELIABILITY OF THE MEAN RESIDENCE TIME CALCULATIONS

The mean residence time calculations constitute the main quantitative analysis of the deintercalation kinetics in the present article. The calculations are based on the method proposed in ref 67 which entails several assumptions. In brief, these assumptions are fulfilled by (a) using a sufficiently large Gaussian deposition time, Δt, to avoid introducing bias in the transition region between two states, (b) ensuring a sufficiently large Gibbs energy barrier between the states to make the transition a rare event, and (c) employing proper CVs that can distinguish the two stable states through significant changes in their values. As mentioned in the article, the selected CVs easily discriminate the transition from the intercalated state due to the abrupt kink in $\alpha(t)$. A practical way to ensure whether the above assumptions are fulfilled is to apply the two-sample Kolmogorov-Smirnov test, which checks whether the distribution of the sampled transition times reflects an exponential distribution corresponding to a Poisson process with the mean residence time we calculated. This hypothesis is considered true if the p-value is greater than 0.05. The mean residence time itself, τ, is extracted by fitting the cumulative distribution function of the simulation data with the cumulative distribution function corresponding to a Poisson process. For each τ, the sum of squared residuals, $R(\tau)$ is computed according to the following equation:

$$R(\tau) = \sum_i [f_{\text{data}}(t_i) - f_{\text{Poisson}}(t_i;\tau)]^2 \quad \text{(S9)}$$

where t_i is the computed residence time in the i-th WT-MetaD run, $f_{\text{data}}(t_i)$ is the cumulative distribution function calculated from the simulations and $f_{exp}(t_i;\tau) = 1 - \exp\left(\frac{t}{\tau}\right)$ the exponential cumulative distribution function corresponding to a Poisson

process with mean residence time τ. Thus, $R(\tau)$ is calculated for many values of τ and the value of τ that minimizes $R(\tau)$ is selected, as $R(\tau)$ quantifies the numerical error of the fitting. It is seen from Figure S8 that the deintercalation in both complexes exhibits clear global minima in their $R(\tau)$. The error in τ is computed via a bootstrap analysis, in which 500 sub-samples from the original simulation data are analyzed. The fitting of the simulation data with exponential cumulative distribution functions is shown in Figure S7.

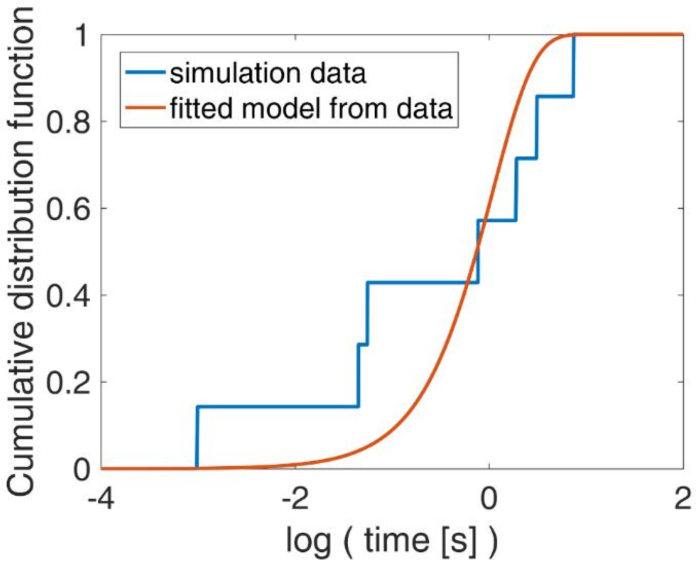

(a)

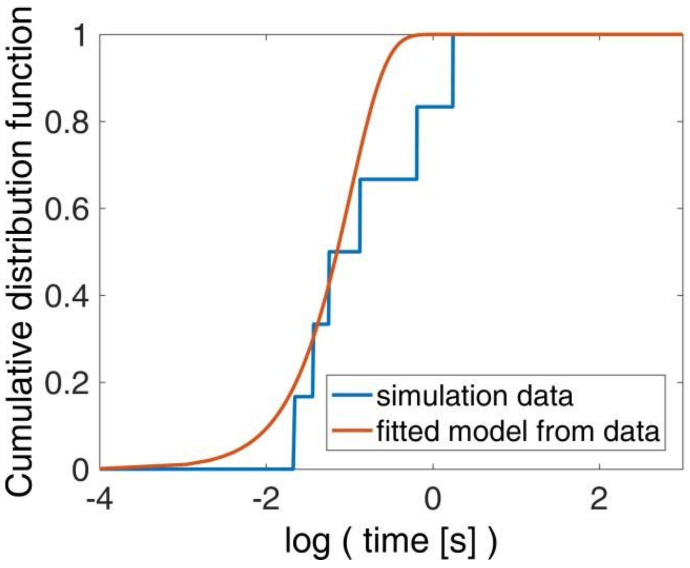

(b)

Figure S7. Fitting of the cumulative distribution function of residence times from the simulation data to the cumulative distribution function of the exponential distribution: (a) 5′-d(ACGTAC|GT)-3′ – berubicin and (b) 5′-d(TGT|ACA)-3′ – berubicin.

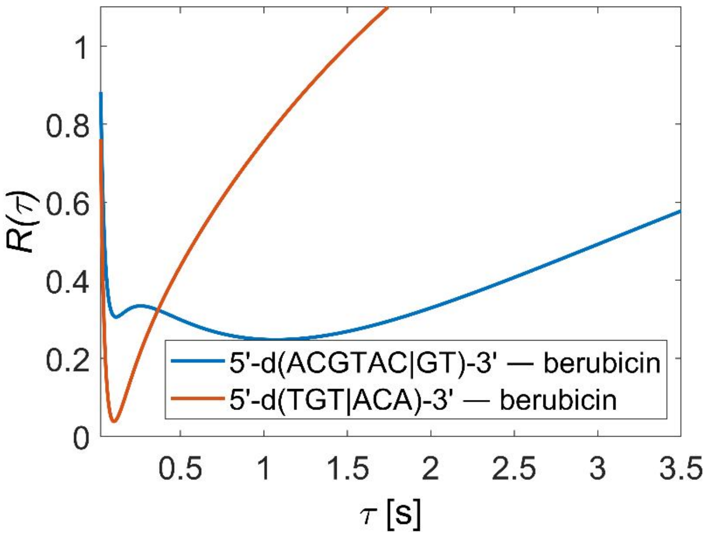

Figure S8. Sum of squared residuals, $R(\tau)$, for 5′-d(ACGTAC|GT)-3′ – berubicin (blue curve) and 5′-d(TGT|ACA)-3′ – berubicin (red curve).

S7. PARAMETERS USED IN PLUMED

After the selection of the CVs, we need to determine the values of the WT-MetaD parameters, namely h_0, Δt, ΔT, σ_X and σ_θ which appear in eq S1. The reader is reminded that h_0 is the initial Gaussian height, Δt is elapsed time between two successive bias additions, ΔT is the temperature factor controlling the convergence rate of the added biases, and σ_X and σ_θ are the standard deviations of X and θ in the multivariate Gaussians, respectively. h_0 must not be set too high, as this could lead to improper sampling. However, it must not be set too low, as this would hinder proper sampling of the CV space. Therefore, a satisfactory selection is a fraction of the thermal energy,[S6] and this was set to h_0=0.25 kJ/mol here. Δt is subject to similar limitations as h_0 regarding its extreme values; it should be large enough to allow the simulation to assimilate the added biases but small enough to ensure a satisfactory sampling rate. The literature on DNA – ligand complexes[29,51,54,55,59] uses values on the order of picoseconds (ps) and thus our choice was 2.0 ps. Regarding ΔT, the literature[S6] mostly discusses the factor $\gamma = \frac{T+\Delta T}{T}$ which is directly related to the decreasing rate of the added biases. In our simulations, γ is set to 17.5. As for σ_X and σ_θ, we need quantitative estimates of the variability of X and θ during a molecular dynamics simulation. To this end, we calculated the standard deviations of X and θ from an unbiased run in the intercalated states of 1 μs duration (taken from ref 11). The values σ_X= 0.25 Å and σ_θ = 2.06° were taken equal to 1/3 of these standard deviations, as suggested by previous studies.[51,S6,S7] It is worth mentioning that we use two kinds of position restraints. The first requirement is to ensure that X remains greater than –1 Å, as we are only interested in the deintercalation through the minor groove, which is the most frequently observed scenario for anthracyclines. The second one is a set of position restraints, applied only in the case of 5′-

d(ACGTAC|GT)-3′. As indicated by the DNA sequence, the intercalation site is near the end of the oligonucleotide sequence. It is well-documented in the literature that the terminal base pairs display fraying effects during molecular dynamics simulations, as mentioned in the main article. Their positional fluctuations might affect the deintercalation process, hence they are kept positionally restrained. The initial configurations are obtained from the unbiased runs in the intercalated state of ref 11.

REFERENCES

(S1) Tiwary, P.; Parrinello, M. A time-independent free energy estimator for metadynamics. *J. Phys. Chem. B* **2015**, *119* (3), 736–742. https://doi.org/10.1021/jp504920s.

(S2) Massey Jr., F. J. The Kolmogorov-Smirnov test for goodness of fit. *J. Am. Sta.t Assoc.* **1951**, *46* (253), 68–78. https://doi.org/10.1080/01621459.1951.10500769.

(S3) Miller, L. H. Table of percentage points of Kolmogorov statistics. *J. Am. Stat. Assoc.* **1956**, *51* (273), 111–121. https://doi.org/10.1080/01621459.1956.10501314.

(S4) Doudou, S.; Burton, N. A.; Henchman, R. H. Standard free energy of binding from a one-dimensional potential of mean force. *J. Chem. Theory Comput.* **2009**, *5* (4), 909–918. https://doi.org/10.1021/ct8002354.

(S5) General, I. J. A note on the standard state's binding free energy. *J. Chem. Theory Comput.* **2010**, *6* (8), 2520–2524. https://doi.org/10.1021/ct100255z.

(S6) Bussi, G.; Branduardi, D. Free-Energy Calculations with Metadynamics: Theory and Practice. In *Reviews in Computational Chemistry Volume 28*; Reviews in Computational Chemistry; 2015; pp 1–49. https://doi.org/https://doi.org/10.1002/9781118889886.ch1.

(S7) Gervasio, F. L.; Laio, A.; Parrinello, M. Flexible docking in solution using metadynamics. *J. Am. Chem. Soc.* **2005**, *127* (8), 2600–2607. https://doi.org/10.1021/ja0445950.
